# *Hansenula polymorpha* Pmt4p Plays Critical Roles in *O*-Mannosylation of Surface Membrane Proteins and Participates in Heteromeric Complex Formation

**DOI:** 10.1371/journal.pone.0129914

**Published:** 2015-07-02

**Authors:** Hyunah Kim, Eun Jung Thak, Dong-Jik Lee, Michael O. Agaphonov, Hyun Ah Kang

**Affiliations:** 1 Department of Life Science, Chung-Ang University, Seoul 156–756, Korea; 2 A.N. Bach Institute of Biochemistry of the Russian Academy of Sciences, Moscow, Russia; CNR, ITALY

## Abstract

*O*-mannosylation, the addition of mannose to serine and threonine residues of secretory proteins, is a highly conserved post-translational modification found in organisms ranging from bacteria to humans. Here, we report the functional and molecular characterization of the *HpPMT4* gene encoding a protein *O*-mannosyltransferase in the thermotolerant methylotrophic yeast *Hansenula polymorpha*, an emerging host for the production of therapeutic recombinant proteins. Compared to the deletion of *HpPMT1*, deletion of another major *PMT* gene, *HpPMT4*, resulted in more increased sensitivity to the antibiotic hygromycin B, caffeine, and osmotic stresses, but did not affect the thermotolerance of *H*. *polymorpha*. Notably, the deletion of *HpPMT4* generated severe defects in glycosylation of the surface sensor proteins HpWsc1p and HpMid2p, with marginal effects on secreted glycoproteins such as chitinase and HpYps1p lacking a GPI anchor. However, despite the severely impaired mannosylation of surface sensor proteins in the *Hppmt4*∆ mutant, the phosphorylation of HpMpk1p and HpHog1p still showed a high increase upon treatment with cell wall disturbing agents or high concentrations of salts. The conditional *Hppmt1pmt4*∆ double mutant strains displayed severely impaired growth, enlarged cell size, and aberrant cell separation, implying that the loss of *HpPMT4* function might be lethal to cells in the absence of HpPmt1p. Moreover, the HpPmt4 protein was found to form not only a homomeric complex but also a heteromeric complex with either HpPmt1p or HpPmt2p. Altogether, our results support the function of HpPmt4p as a key player in *O*-mannosylation of cell surface proteins and its participation in the formation of heterodimers with other PMT members, besides homodimer formation, in *H*. *polymorpha*.

## Introduction

Protein *O*-mannosylation is an essential protein modification that is evolutionarily conserved from bacteria and yeast to humans and is initiated by protein *O*-mannosyltransferases (Pmts) [1]. In the endoplasmic reticulum (ER), Pmt proteins catalyze the transfer of mannose residue to Ser/Thr residues of target proteins [2]. Seven Pmt isoforms (ScPmt1–7p), which are ER membrane proteins comprising multiple transmembrane domains, have been identified in the baker’s yeast *Saccharomyces cerevisiae* [2,3]. The *S*. *cerevisiae* Pmt isoforms are grouped into three major subfamilies referred to as PMT1 (ScPmt1/5/7p), PMT2 (ScPmt2/3/6p), and PMT4 (ScPmt4p) [4,5]. Orthologs of *S*. *cerevisiae* Pmt proteins have been reported in many other yeast and filamentous fungal species, including *Aspergillus fumigatus* [6], *Aspergillus nidulans* [7], *Botrytis cinerea* [8], *Candida albicans* [9], *Cryptococcus neoformans* [10], *Pichia pastoris* [11], *Schizosaccharomyces pombe* [12,13], *Trichoderma reesei* [14], and *Ustilago maydis* [15]. Moreover, PMT homologs are present in higher multicellular eukaryotes such as *Drosophila melanogaster*, zebrafish, mice, and humans. In *D*. *melanogaster*, only two PMT family members, *rotated abdomen* and *twisted*, are present [16,17]. The same is true in the case of zebrafish, mice, and humans, which possess POMT1 (a member of the PMT4 subfamily) and POMT2 (a member of the PMT2 subfamily) [17–19].

Although PMT family members show a high degree of conservation, several significant distinctive features are present between PMT1/PMT2 and PMT4 subfamily members. First, the PMT1/PMT2 subfamilies are highly redundant, whereas the PMT4 subfamily has only one representative per species [20]. Second, despite the fact that all PMT family members share three conserved sequence motifs, A, B, and C, these conserved motifs show significant variation between PMT1/PMT2 and PMT4 subfamily members [20]. Third, PMT1 and PMT2 subfamily members function together as heterodimers for maximum activity, whereas the unique representative of the PMT4 subfamily forms a homodimer in fungi [3]. However, a very recent study demonstrated that *A*. *nidulans* PmtA (subfamily PMT2) and PmtB (subfamily PMT1) can also form heterodimers with PmtC (subfamily PMT4) [21], which is similar to the POMT1-POMT2 complex formation in humans [22]. Furthermore, PMT1/PMT2 and PMT4 subfamily members modify different acceptor protein substrates *in vivo*. For example, Kre9p, Bar1p, Pir2p/Hsp150p, and Aga2p are exclusively *O*-mannosylated by Pmt1p-Pmt2p complexes in *S*. *cerevisiae* [23]. In contrast, Kex2p, Gas1p [23], Axl2p [24], Fus1p [25], and β-amyloid precursor protein (APP) [26] are *O*-mannosylated by Pmt4p. The WSC family members as well as Mid2p [27], Mtl1p [28], and Ccw5p/Pir4p [29] are mannosylated by both complexes, but in distinct regions. It was reported that the preferred substrates of Pmt4p are membrane-associated proteins bearing Ser/Thr-rich domains in *S*. *cerevisiae* [30].

*Hansenula polymorpha* is a thermotolerant methylotrophic yeast that can grow rapidly using methanol as its sole source of carbon and energy, and at high temperatures up to 48°C [31]. This yeast has been developed as a favorable host for the production of recombinant pharmaceuticals [32], since it has several important advantages as a versatile cell factory. It contains several strong and inducible promoters derived from genes of the methanol metabolic pathway, specifically methanol oxidase (*MOX*), dihydroxyacetone synthase (*DHAS*) and formate dehydrogenase (*FMDH*) [33,34]. Furthermore, *H*. *polymorpha* does not hyperglycosylate secreted proteins, which often causes a problem in heterologous protein production in *S*. *cerevisiae* [35,36]. Systematic studies on the *H*. *polymorpha* genes involved in *N*-linked glycosylation facilitated the development of glycoengineered *H*. *polymorpha* strains for the production of human-type *N*-glycans [37–40]. Our recent study reported that *H*. *polymorpha* has five *PMT* genes (*HpPMT1*, *HpPMT2*, *HpPMT4*, *HpPMT5*, and *HpPMT6*) encoding protein *O*-mannosyltransferases [41]. Lack of HpPmt1p results in increased temperature sensitivity and decreased *O*-glycosylation of the chitinase protein [41,42]. The *Hppmt1pmt5*∆ and *Hppmt1pmt6*∆ double mutants showed increased sensitivity to cell wall stressors and aberrant *O*-mannosylation of the surface glycoproteins HpWsc1p and HpMid2p [41]. Here, we report the functional and molecular characterization of the *H*. *polymorpha PMT4* gene, which is the only member of the PMT4 subfamily. We present a line of evidence supporting our conclusion that HpPmt4p plays a critical role essentially in the *O*-mannosylation of surface membrane proteins. Moreover, we show that Pmt4p forms not only a homodimeric complex but also forms heterodimeric complexes either with Pmt1p or Pmt2p in *H*. *polymorpha*.

## Materials and Methods

### Yeast strains, plasmids, primers, and culture conditions

The *H*. *polymorpha* strains and plasmids used in this study are listed in Table 1 and S1 Table, respectively. The primers used for the construction of plasmids and strains are listed in S2 Table. Yeast cells were grown at 37°C in YPD medium (1% yeast extract, 2% Bacto peptone, and 2% glucose). The transformation of *H*. *polymorpha* was performed according to the modified lithium acetate-dimethyl sulfoxide method [43]. A selective synthetic complete (SC) medium (0.67% yeast nitrogen base without amino acids, 2% glucose, 0.77 g/L drop-out amino acid supplement without uracil or leucine) or YPD containing 80 μg/mL zeocin (Invitrogen) or 100 μg/mL hygromycin B (Sigma) was used for the selection of various yeast transformants.

**Table 1:** List of *H*. *polymorpha* strains used in this study.

Strain	Description	Reference
1B	A derivative strain of CBS4732, *ade2 leu2*	[42]
1BQ-u	*ade2 leu2 URA3*::*ADE2 mox*::*uPAQ302*	this study
1BQ-LA (wild-type)	*ade2 leu2 mox*::*uPAQ302[ADE2][LEU2]*	this study
1BQ-u-*pmt1* (*Hppmt1*∆)	1BQ-u *pmt1*::*LEU2*	this study
1BQ-u-*pmt4* (*Hppmt4*∆)	1BQ-u *pmt4*::*LEU2*	this study
1BQ-u-*pmt1*/P_*MET3*_-*PMT4 (Hppmt1pmt4*∆)	1BQ-u *pmt1*::*LEU2 pmt4*::P_*MET3*_-*PMT4*	this study
1BQ-LA/WSC1H	1BQ-LA [pHINZ-HpWSC1H]	this study
1BQ-u-*pmt1*/WSC1H	1BQ-u *pmt1*::*LEU2* [pHINZ-HpWSC1H]	this study
1BQ-u-*pmt4*/WSC1H	1BQ-u *pmt4*::*LEU2* [pHINZ-HpWSC1H]	this study
1BQ-u-*pmt4*/WSC1H/PMT4F	1BQ-u *pmt4*::*LEU2* [pHINZ-HpWSC1H] [pDUN-HpPMT4F]	this study
1BQ-LA/MID2F	1BQ-LA[pHINZ-HpMID2F]	this study
1BQ-u-*pmt1*/MID2F	1BQ-u *pmt1*::*LEU2* [pHINZ-HpMID2F]	this study
1BQ-u-*pmt4*/MID2F	1BQ-u *pmt4*::*LEU2* [pHINZ-HpMID2F]	this study
1BQ-u-*pmt4*/MID2F/PMT4H	1BQ-u *pmt4*::*LEU2* [pHINZ-HpMID2F] [pHINZU-HpPMT4H]	this study
1BQ-u/YPS1H	1BQ-u [pDLUMOX-HpYPS1H]	this study
1BQ-u-*pmt1*/YPS1H	1BQ-u *pmt1*::*LEU2* [pDLUMOX-HpYPS1H]	this study
1BQ-u-*pmt4*/YPS1H	1BQ-u *pmt4*::*LEU2* [pDLUMOX-HpYPS1H]	this study
1BQ-u-*pmt4*/PMT4H	1BQ-u *pmt4*::*LEU2* [pHINZU-HpPMT4H]	this study
1BQ-u-*pmt4*/PMT4F	1BQ-u *pmt4*::*LEU2* [pDUN-HpPMT4F]	this study
1BQ-u-*pmt4*/ PMT4H/PMT4F	1BQ-u *pmt4*::*LEU2* [pDUN-HpPMT4F] [pHINZ-HpPMT4H]	this study
1BQ-u-*pmt1*/PMT1H	1BQ-u *pmt1*::*LEU2* [pHINZ-HpPMT1H]	this study
1BQ-LA/PMT2F	1BQ-LA [pHIHT-HpPMT2F]	this study
1BQ-u-*pmt1* /PMT1H/PMT4F	1BQ-u *pmt1*::*LEU2* [pHINZ-HpPMT1H] [pDUN-HpPMT4F]	this study
1BQ-u-*pmt4*/PMT2F	1BQ-u *pmt4*::*LEU2* [pHIGAZ-HpPMT2F]	this study
1BQ-u-*pmt4*/ PMT2F/PMT4H	1BQ-u *pmt4*::*LEU2* [pHIGAZ-HpPMT2F] [pHINZU-HpPMT4H]	this study
DL1-L/PMT4H	*leu2* [pHINZ-HpPMT4H]	this study
DL1-L/PMT4F	*leu2* [pHINHT-HpPMT4F]	this study
DLM-20/PMT1H	*leu2 pmt1*::*LEU2* [pHIGAZ-HpPMT1H]	[41]
DL1-L/PMT2F	*leu2* [pHIHT-HpPMT2F]	[41]
DLM-20/PMT1H/PMT4F	*leu2 pmt1*::*LEU2* [pHIGAZ-HpPMT1H] [pHINHT-HpPMT4F]	this study
DL1-L/PMT2F/PMT4H	*leu2* [pHIHT-HpPMT2F] [pHINZ-HpPMT4H]	this study

### Construction of *pmt* mutants

The 1BQ-u strain was constructed in two steps from the 1B strain, a derivative of *H*. *polymorpha* CBS4732 (*Ogataea polymorpha*) [44]. First, the *MOX* ORF was replaced with the N302Q mutant of the ORF encoding human urokinase-type plasminogen activator [45]; then, in the 1BQ strain obtained, the *URA3* gene was disrupted with the *ADE2* selectable marker. The *HpPMT1* gene in the 1BQ-u strain was disrupted using the pSS18-derived *HpPMT1* disruption cassette, which was constructed in a previous study [42], generating the *Hppmt1*∆ mutant strain (1BU-u-*pmt1*). The *HpPMT4* disruption cassette was constructed by introducing inverse recombination arms into a *HpLEU2*-equipped integrative vector according to a previously reported approach [46]. Briefly, *H*. *polymorpha* CBS4732 genomic DNA was digested with *Hind*III, self-ligated, and used as a template for PCR with the primers HpPMT4D_F and HpPMT4D_R. The PCR product containing inverse recombination arms was digested with *Eco*RI and *Xho*I for cloning between the *Eco*RI and *Sal*I sites of the pCHLX plasmid [47]. The resulting plasmid pCSS27 was digested with *Hind*III to obtain the *HpPMT4* disruption cassette.

The plasmid pDUM3P600-HpPMT4D, used to construct the *H*. *polymorpha pmt1pmt4*∆ double mutant strain bearing an *HpPMT4* allele under the control of the *HpMET3* promoter (1BQ-u-*pmt1*/P_*MET3*_-*PMT4*), was obtained as follows: The 600-bp *HpMET3* promoter and the 750-bp partial 5′ ORF region of *HpPMT4* were PCR amplified from the *H*. *polymorpha* CBS4732 wild-type genomic DNA using the *Sal*I-HpMET3_F/HpMET3_R-*Eco*RI and *Eco*RI-HpPMT4D_F/HpPMT4D-*Cla*I_R primer pairs, respectively. The *Sal*I/*Eco*RI-digested *HpMET3* promoter was subcloned into *Sal*I/*Eco*RI sites of pDUMOX-msdS (HA-HDEL) [37], generating pDUM3P600-msdS. Subsequently the *Eco*RI/*Cla*I-digested *HpPMT4* fragment was cloned into *Eco*RI/*Cla*I-digested pDUM3P600-msdS, resulting in pDUM3P600-HpPMT4D, which possessed the incomplete *HpPMT1* ORF under control of the *HpMET3* promoter. The *Kpn*I-digested plasmid pDUM3P600-PMT4D was integrated into the *HpPMT4* locus of the 1BU-u-*pmt1* by a single crossover recombination. The correct integration was verified by PCR.

### Construction of expression vectors and strains for epitope-tagged Pmt4 proteins

To construct an expression vector for FLAG-tagged HpPmt4p, the DNA fragment containing the 523-bp promoter and full-length ORF without a stop codon of *HpPMT4* was PCR amplified from *H*. *polymorpha* CBS4732 wild-type genomic DNA using the primer pair *Nhe*I-HpPMT4_F/HpPMT4_R-*Asc*I. The PCR product was digested with *Nhe*I/*Asc*I and ligated into the *Nhe*I/*Asc*I-digested pHIGAHT-4FLAG [41], resulting in the plasmid pHINHT-HpPMT4F. The 2.9 kb *Nhe*I/*Cla*I fragment containing the *HpPMT4* promoter and ORF tagged with four units of FLAG was obtained from pHINHT-HpPMT4F and ligated with the *Cla*I/*Sal*I-digested pDUMOX-MsdS (HA-HDEL) [37], generating an *H*. *polymorpha* expression vector for a FLAG-tagged HpPmt4p, pDUN-HpPMT4F. To generate an *Hppmt4*∆ strain expressing a FLAG-tagged HpPmt4p (*Hppmt4*∆/PMT4F), the circular plasmid pDUN-HpPMT4F was introduced into the *Hppmt4*∆ strain.

To construct an expression vector for HA-tagged HpPmt4p, the 2.7 kb fragment containing the *HpPMT4* promoter and ORF was digested with *Nhe*I/*Asc*I from pHINHT-HpPMT4F and ligated with the *Nhe*I/*Asc*I-digested pHIGAZ-6HA [41], resulting in plasmid pHINZ-HpPMT4H. The *HpURA3* gene fragment was excised from pTHpURA3-LZ [48] by *Bam*HI and inserted into the *Bgl*II-digested pHINZ-HpPMT4H, generating pHINZU-HpPMT4H. The *Dra*I-digested pHINZU-HpPMT4H or pHINZ-HpPMT4H was integrated by homologous recombination into the *HpPMT4* promoter locus of the *Hppmt4*∆ or *Hppmt4*∆/PMT4F strains, respectively, generating an *H*. *polymorpha* strain expressing HA-tagged HpPmt4p (*Hppmt4*∆/PMT4H) and another co-expressing HA-tagged HpPmt4p and FLAG-tagged HpPmt4p (*Hppmt4*∆/PMT4F/PMT4H), respectively. To generate strains co-expressing either HA-tagged HpPmt1p and FLAG-tagged HpPmt4p (DLM-20/PMT1H/PMT4F) or FLAG-tagged HpPmt2p and HA-tagged HpPmt4p (DL1-L/PMT2F/PMT4H), the *Xho*I-digested pHINHT-HpPMT4F or the *Eco*RI-digested pHINZ-HpPMT4H was integrated by homologous recombination into the *HpPMT4* locus of the DLM-20/PMT1H or DL1-L/PMT2F strain [41], respectively. The vectors pHINZ-HpPMT4H and pHINHT-HpPMT4F were individually introduced into the wild-type strain of DL-1 background in the same way, generating *H*. *polymorpha* strains expressing HA-tagged HpPmt4p (DL1-L/PMT4H) and FLAG-tagged HpPmt4p (DL1-L/PMT4F), respectively.

To construct an expression vector for HA-tagged HpPmt1p, pHINZ-HpPMT1H, the PCR fragment containing the full-length ORF without the stop codon of the *HpPMT1* gene was obtained from *H*. *polymorpha* CBS4732 genomic DNA using the primer pair *Bgl*II-HpPMT1_F/HpPMT1_R-*Xho*I. The PCR product was cloned as a *Bgl*II/*Xho*I-digested fragment into pHIGAZ-HpPMT1H [41], generating pHINZ-HpPMT1H, which was cut at the *Pst*I site within the *HpPMT1* ORF and integrated by homologous recombination into the genomic *HpPMT1* locus of the 1BU-u-*pmt1* (*Hppmt1*∆) strain, resulting in a *Hppmt1*∆/PMT1H strain. For the construction of an *H*. *polymorpha* wild-type strain expressing a FLAG-tagged HpPmt2p strain (1BQ-LA/PMT2F), the plasmid pHIHT-HpPMT2F [41] was cut at the *Ava*I site within the *HpPMT2* ORF and integrated by homologous recombination into the genomic *HpPMT2* locus of the 1BQ-LA strain. For construction of a *H*. *polymorpha* 1BU-u-*pmt4* (*Hppmt4*∆) mutant strain expressing a FLAG-tagged HpPmt2p (*Hppmt4*∆/PMT2F), pHIGAZ-HpPMT2F [41] was cut at the *Eco*RI site within the *HpPMT2* ORF for linearization and integrated by homologous recombination into the genomic *HpPMT2* locus of the *Hppmt4*∆ strain. To generate strains co-expressing either HA-tagged HpPmt1p and FLAG-tagged HpPmt4p (*Hppmt1*∆/PMT1H/PMT4F) or FLAG-tagged HpPmt2p and HA-tagged HpPmt4p (*Hppmt4*∆/PMT2F/PMT4H) in the CBS4732 strain background, the circular plasmid pDUN-HpPMT4F or the *Eco*RI-digested pHINZ-HpPMT4H were integrated in the *Hppmt1*∆/PMT1H or the *Hppmt4*∆/PMT2F strain, respectively.

### Construction of expression vectors and strains for epitope-tagged glycoproteins

For the construction of pHINZ-HpWSC1H, an expression vector of the HA-tagged HpWsc1p (HpWsc1p^HA^), the DNA fragment containing the 500-bp promoter and the full-length ORF without the stop codon of *HpWSC1* was amplified by PCR from CBS4732 genomic DNA using the primer pair *Bgl*II-HpWSC1_pm/HpWSC1_R-*Asc*I. The PCR product was digested with *Bgl*II/*Asc*I and ligated with *Bgl*II/*Asc*I-digested pHIGAZ-6HA, generating pHINZ-HpWSC1H. To construct plasmid pHINZ-HpMID2F, an expression vector of FLAG epitope-tagged HpMid2p (HpMid2p^FLAG^), the PCR fragment containing the 500-bp promoter and the full-length ORF without the stop codon of the *HpMID2* gene was obtained using the primer pair *Bgl*II-HpMID2_pm/HpMID2_R-*Asc*I. The PCR product was cloned as a *Bgl*II/*Asc*I-digested fragment into pHIGAZ-4FLAG digested with *Bgl*II and *Asc*I enzymes, generating pHINZ-HpMID2F. The *Eco*RI-digested pHINZ-HpWSC1H or *Sca*I-digested pHINZ-HpMID2F was integrated by homologous recombination into the genomic *HpWSC1* or *HpMID2* locus of the wild-type, *Hppmt1*∆, and *Hppmt4*∆ strains, respectively. For complementation of the *Hppmt4*∆ mutants expressing either HpWsc1p^HA^ or HpMid2p^FLAG^ by reintroduction of *HpPMT4*, the *Hppmt4*∆/MID2F and *Hppmt4*∆/WSC1H strains were transformed with the *Dra*I-digested pHINZU-HpPMT4H or the circular plasmid pDUN-HpPMT4F, respectively. To generate pDLUMOX-HpYPS1H, the *lacZ-HpURA3-lacZ* cassette was excised from pTHpURA3-LZ digested with *Eco*RI, treated with the Klenow fragment, and inserted into pMOX-YPS1ct-His [49] at the unique *Psi*I site. The resulting circular plasmid pDLUMOX-HpYPS1H was transformed into wild-type, *Hppmt1*∆, and *Hppmt4*∆ strains.

### MAP kinase phosphorylation analysis

To detect the expression of phospho-Mpk1, the *H*. *polymorpha* strains were grown to an OD_600_ of 1.0 in YPD medium at 37°C, and then one-half of the culture was harvested, while the second half was treated with 0.05% SDS, 2 μg/mL caspofungin (CAS), 2.5 μg/mL tunicamycin (TM), 0.2 mg/mL calcofluor white (CFW), 20 mM caffeine, 10 mg/mL Congo red (CR), or 0.5 M sodium chloride for 2 hr. The cells were harvested and lysed by vortexing with glass beads (425–600 μm in diameter, Sigma) in phosphatase inhibitor lysis buffer [41]. Supernatants were separated by centrifugation at 2,500 × *g* for 5 min at 4°C and analyzed with 10% SDS-PAGE. For detection of phosphorylated Mpk1 and phospho-Hog1 proteins, the anti-phospho-p44/p42 MAPK and anti-phospho-p38 MAPK antibodies (Cell Signaling Technology) were used, respectively, and anti-ScHog1 antibody (Santa Cruz Biotechnology) was used to detect Hog1 protein. The anti-beta actin antibody (Abcam) was used as a loading control.

### Preparation of membrane fractions and co-immunoprecipitation

Membrane fractions were isolated as previously described by Kim *et al*. [41]. HpPmt4p^HA^ protein was immunoprecipitated from sodium deoxycholate (SDC) extract containing 300 μg of proteins by using the ProFound HA Tag IP/Co-IP Kit (Thermo Scientific). Similarly, HpPmt4p^FLAG^ protein was immunoprecipitated from SDC extract containing an equal amount of protein in the final volume of 1 mL of lysis buffer using the FLAG-Tagged Protein IP Kit (Sigma).

### Microscopy analysis

Yeast cells were grown to an OD_600_ of ~2 in SD medium, exposed to 2 mM cysteine for 24 hr, and harvested. Cells were adjusted to a final concentration of 1 × 10^7^ cells/mL, fixed with 3.7% formaldehyde in a rotator for 10 min at RT, and washed with 1x PBS buffer three times. For cell wall material staining, cells were stained with fluorescent brightener 28 (CFW; Sigma Aldrich) at a final concentration of 5 mg/mL in a rotator for 10 min and washed with 1x PBS buffer three times. Light and fluorescence microscopy pictures were taken using a Zeiss LSM700 confocal microscope equipped with Axio Observer. Images were processed with ZEN2011 software (Zeiss).

## Results

### Construction and growth phenotype analysis of an *Hppmt4* null mutant strain

**Fig 1:**
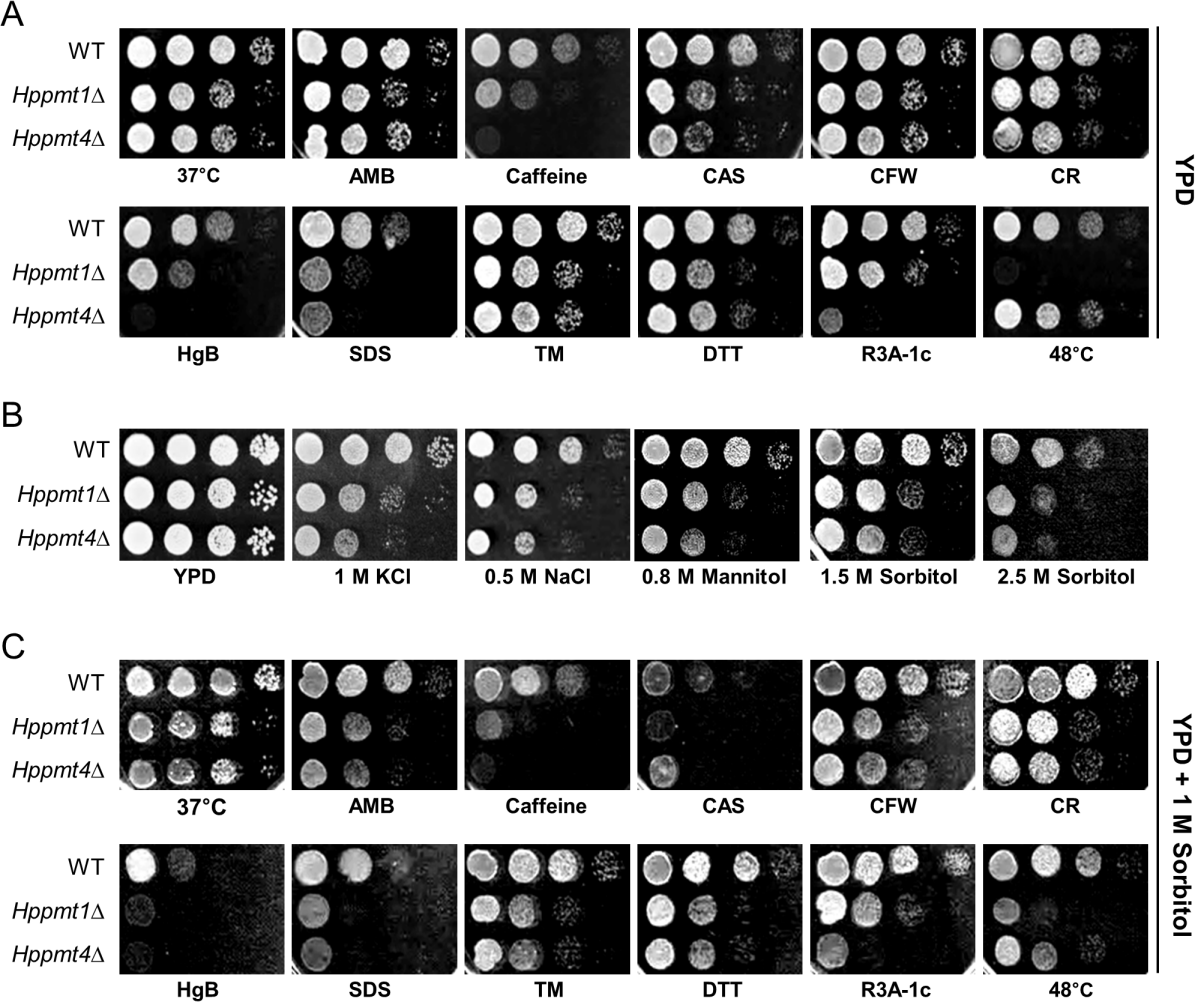
Growth phenotype analysis of *H*. *polymorpha pmt* mutant strains. (A) *H*. *polymorpha* cells were spotted on YPD only plates or YPD plates containing 1 μg/mL amphotericin B (AMB), 10 mM caffeine, 0.3 μg/mL caspofungin (CAS), 0.1 mg/mL calcofluor white (CFW), 3 mg/mL Congo red (CR), 5 μg/mL hygromycin B (HgB), 0.01% SDS, 0.5 μg/mL tunicamycin (TM), 10 mM dithiothreitol (DTT) or 45 μM Pmt1p inhibitor R3A-1c, or on a YPD plate cultivated at 48°C. (B) *H*. *polymorpha* cells were spotted on YPD plates supplemented with NaCl, KCl, mannitol, or sorbitol at indicated concentrations. (C) *H*. *polymorpha* cells were cultivated on YPD plates containing indicated stress reagents in the presence of 1 M sorbitol. The overnight cultivated wild-type 1BQ-LA and *Hppmt* mutant cells were adjusted to an OD_600_ of 1.0, diluted by 10-fold serial dilutions down to a 10^−4^ dilution, and then spotted onto YPD plates supplemented with various stress reagents at indicated concentrations. Plates were incubated for 2 days.

To obtain information on the function of *PMT4* in *H*. *polymorpha*, we constructed an *Hppmt4* null mutant strain in the CBS4732 derivative and analyzed its growth phenotypes. The sensitivity of the *Hppmt4*Δ mutant strain to various cell stresses, as well as antibiotic and antifungal drugs, was analyzed and compared to that of the *Hppmt1*∆ mutant strain containing a deletion of *HpPMT1* (Fig 1A). The growth of the *Hppmt1*∆ and *Hppmt4*Δ mutant strains was significantly inferior to that of the wild-type strain in the presence of cell wall destabilizers such as CAS, CR, or SDS. However, the *H*. *polymorpha pmt* mutants showed marginal sensitivity to antifungals, such as amphotericin B (AMB) and CFW, and to an ER stress inducer TM, an inhibitor of Asn-linked glycosylation causing ER stress [50]. The retarded growth of both *pmt* mutants was more apparent in the presence of another ER stress inducer DTT, an inhibitor of disulfide bond formation [51], than in the presence of TM. Notably, the *Hppmt4*Δ mutant strain was more susceptible to cell wall stressors, such as caffeine and the antibiotic hygromycin B (HgB), and to the Pmt1p inhibitor R3A-1c, than the *Hppmt1*Δ strain. At elevated temperatures, the growth of the *Hppmt4*Δ mutant was marginally decreased, whereas the *Hppmt1*Δ strain displayed a profound temperature-sensitive growth defect. Such increased sensitivities of the *Hppmt4*Δ mutant strain to cell wall stressors and at high temperatures were also consistently observed in another independent *Hppmt4*Δ clone (S1A Fig). Altogether, these phenotypes suggest an important role for *HpPMT4* in maintaining cell wall integrity. It is noteworthy that the *Hppmt* mutant strains displayed increased susceptibility to various salt stresses (Fig 1B). When incubated at high salt concentrations, such as 1 M KCl or 0.5 M NaCl, the *Hppmt1*∆ and *Hppmt4*∆ mutant strains grew significantly slower than the wild-type strain. Interestingly, the *Hppmt4*∆ mutant appeared to be more susceptible to high concentrations of salts than the *Hppmt1*∆ strain. It is notable that the growth of the *Hppmt1*∆ and *Hppmt4*∆ mutants of the *H*. *polymorpha* CBS4732 strain was inhibited even by sorbitol and mannitol, which are known as osmotic stabilizers. For that reason, the increased sensitivity to cell wall disturbing reagents of the *Hppmt1*Δ and *Hppmt4*Δ mutants could not be complemented by the addition of 1 M sorbitol (Fig 1C). The supplementation with other osmotic protectors, such as 1 M glycerol or 1 M sucrose, also did not recover the growth defects of *Hppmt1*∆ and *Hppmt4*∆ mutants at high temperatures and in the presence of cell wall stressors (S1B and S1C Fig). Taken together, these results suggest that the loss of HpPmt4p results in significant alteration of cell wall integrity, generating increased sensitivity to cell wall and osmotic stresses.

### *O*-mannosylation analysis of glycoproteins in *Hppmt4*∆ mutants

**Fig 2:**
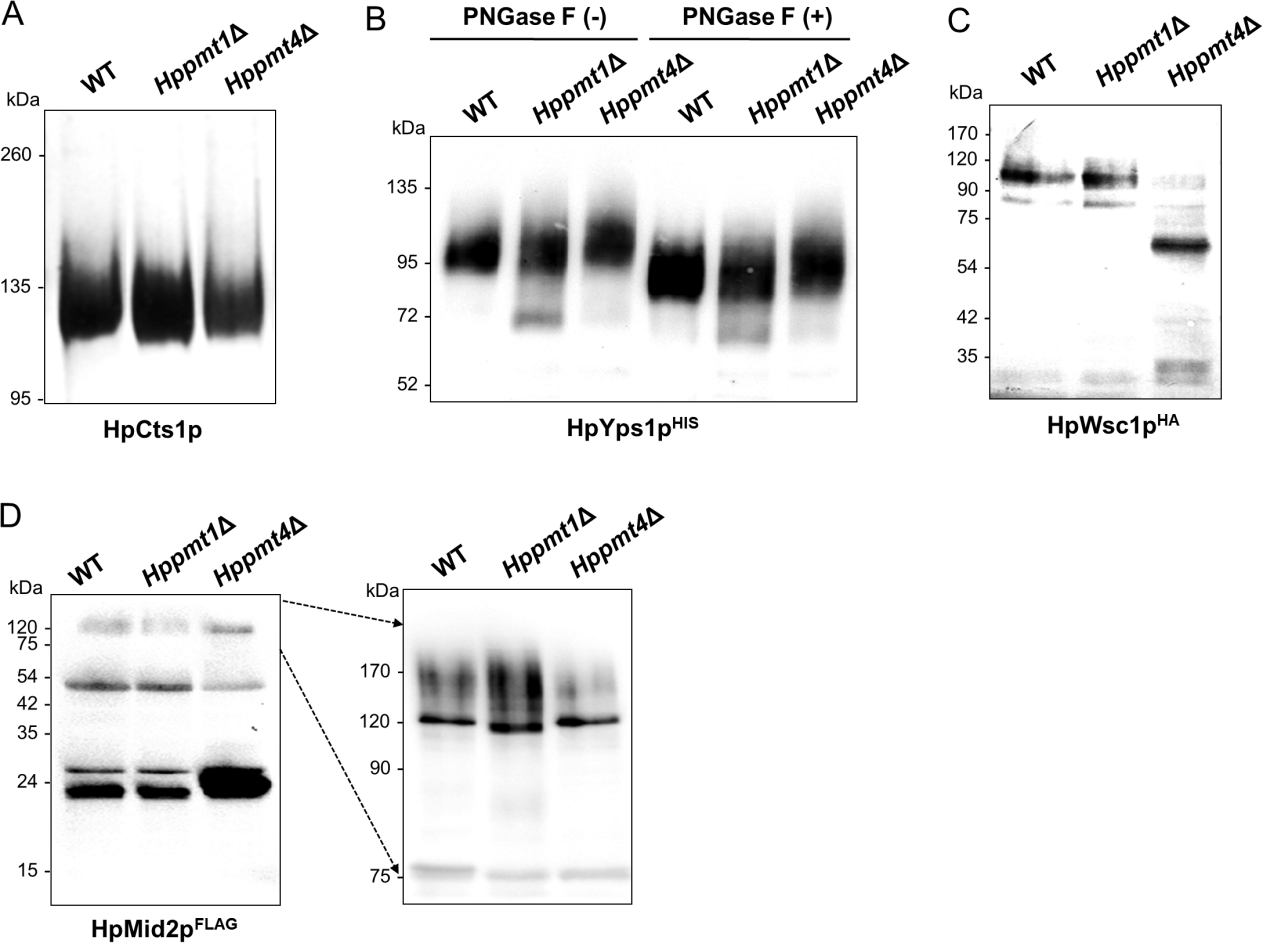
Analysis of *O*-mannosylation of glycosylated proteins in the *H*. *polymorpha* wild-type and *Hppmt* mutant strains. (A) Chitinase was isolated from culture media of *H*. *polymorpha* wild-type 1BQ-LA (WT), *Hppmt1*∆, and *Hppmt4*∆ mutant strains by binding to chitin beads, and analyzed by 6% SDS-PAGE. (B) C-terminally truncated HpYps1 proteins expressed in the *H*. *polymorpha* wild-type, *Hppmt1*∆, and *Hppmt4*∆ mutant strains weredetected using anti-His antibodies with (+) and without (-) PNGase F treatment. (C) Cell extracts from the *H*. *polymorpha* wild-type and from several *pmt* mutants expressing HpWsc1p^HA^ were subjected to 8% SDS-PAGE followed by western blot analysis with anti-HA antibody. (D) HpMid2p^FLAG^ expressed in the wild-type, *Hppmt1*∆, and *Hppmt4*∆ mutant strains was resolved by 6% SDS-PAGE (left panel) and 12% SDS-PAGE (right panel). Blots were sequentially probed with anti-FLAG antibody. The same samples were loaded in each panel.

To investigate the influence of *Hppmt4* mutation on *O*-glycosylation activity *in vivo*, the electrophoretic mobility of the endogenous chitinase, an extracellular glycoprotein that is exclusively *O*-glycosylated without *N*-glycosylation [37,42], was analyzed. While the chitinase of the *Hppmt1*∆ mutant migrated slightly faster than that of the wild-type strain, no apparent change was observed in the electrophoretic mobility of chitinase of the single *Hppmt4*∆ mutant (Fig 2A), suggesting that Pmt4p is not involved in *O*-mannosylation of chitinase in *H*. *polymorpha*. We further investigated effects of *pmt4* and *pmt1* mutations on glycosylation patterns of secreted His-tagged Yps1p (HpYps1p^HIS^), which lacks a GPI anchor. Whereas HpYps1p is an aspartic protease largely localized on cell surface via GPI, HpYps1p^HIS^ having a deletion of a GIP signal (deletion from ω+4 position to C-terminus) is efficiently secreted as a soluble form [49]. The hyperglycosylated forms of HpYps1p^HIS^ were detected by immunoblot analysis, using an anti-His antibody (Fig 2B). After cleavage of the signal peptides, the molecular weight (MW) of secreted HpYps1p^HIS^ was predicted to be approximately 55 kDa, while its *N*-glycosylated form in the ER is ~75 kDa. The secreted HpYps1p^HIS^ displayed a single broad band of higher MW (~90–110 kDa) due to the addition of *O*-mannosylation in the wild-type strain. An underglycosylated form of HpYps1p^HIS^ of ~70 kDa was apparently observed as a minor fraction in the *Hppmt1*Δ mutant, whereas the underglycosylated form of HpYps1p^HIS^ was barely detected in the *Hppmt4*Δ mutant as a faint smear. After PNGase F treatment, which removes *N*-linked glycans from glycoproteins, the differential migration patterns of secreted HpYps1p^HIS^ in the wild-type and *Hppmt* mutant strains were still seen, indicating that the difference in electrophoretic mobility is caused by differential *O*-glycosylation. Thus, this suggested that the secreted form of C-terminally truncated Yps1p is preferentially modified by Pmt1p, although Pmt4p may also be partly involved in the modification of Yps1p in *H*. *polymorpha*. We then examined whether HpPmt4p is required in *O*-mannosyl modification of cell surface glycoproteins, such as Wsc1p and Mid2p. For this analysis, either HpWsc1p with six copies of a C-terminal HA tag (HpWsc1p^HA^) or HpMid2p with four copies of the C-terminal FLAG tag (HpMid2p^FLAG^) was expressed in the *H*. *polymorpha* wild-type and *pmt* mutant strains. Although the deduced MW of HpWsc1p^HA^ is 41.8 kDa, HpWsc1p^HA^ migrated on SDS-PAGE with an apparent MW of approximately 100 kDa in the wild-type strain due to intensive *O*-linked mannosylation (Fig 2C). In the *Hppmt1*∆ mutant, we did not detect any significant difference in the migration pattern of HpWsc1p^HA^ compared to that for the wild-type cells. In contrast, we clearly observed a remarkable reduction in the MW of HpWsc1p^HA^ in the *Hppmt4*∆ mutant. We detected an abundant form of HpWsc1p^HA^ at 72 kDa, which was not observed for the wild-type and *Hppmt1*∆ mutant strains (Fig 2C). Moreover, we observed a band of HpWsc1p^HA^ smaller than 35 kDa, which is assumed to be the aberrant proteolytic product of unmodified HpWsc1p [27]. A similar pattern of MW change due to a lack of *O*-mannosylation was obtained for HpMid2p^FLAG^ in the *Hppmt4*∆ mutant strain. In the wild-type strain, smeared bands of HpMid2p^FLAG^ of approximately 120–200 kDa, which is much larger than the predicted size of 46.8 kDa, were detected by anti-FLAG antibody. In the *Hppmt1*Δ mutant, the levels of the high MW forms of HpMid2p^FLAG^ were slightly lower than those in the wild-type cells (Fig 2D, left panel). However, in the absence of HpPmt4p, the total amount of these high MW forms was dramatically decreased while small fragments of HpMid2p^FLAG^ were generated (Fig 2D, right panel). The re-introduction of functional HpPmt4p in the *Hppmt4*∆ mutant strains expressing HpWsc1p^HA^ or HpMid2p^FLAG^ were shown to completely restore the altered *O*-mannosylation patterns and thereby the instability of HpWsc1 and HpMid2 proteins to those observed in the wild-type strains (S2 Fig). From these data, we conclude that HpPmt4p plays a major role exclusively in *O*-mannosylation of membrane associated proteins, which is critical for maintaining the stability of these membrane proteins.

### Analysis of MAP kinase activation in the *Hppmt4*∆ mutant

**Fig 3:**
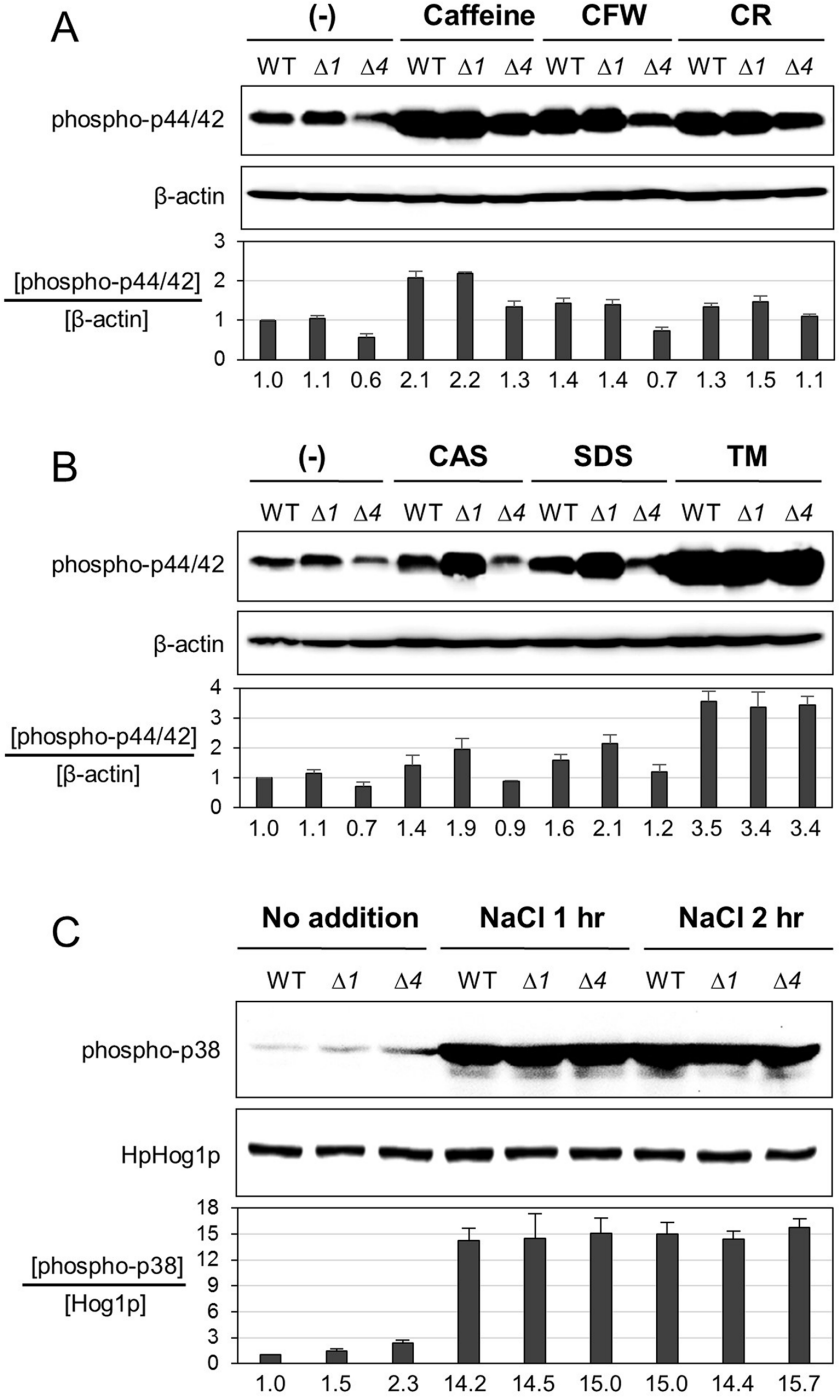
Analysis of activation of the cell wall integrity pathway and HOG pathway in *H*. *polymorpha pmt* mutant strains. (A and B) Analysis of MAP kinase activation. The total soluble protein samples were obtained from the wild-type 1BQ-LA (WT), *Hppmt1*∆ (*∆1*), and *Hppmt4∆* (*∆4*) mutant strains, which were grown to early exponential phase and then treated for 2 hr with 20 mM caffeine, 0.2 mg/mL CFW, 10 mg/mL CR, 2 μg/mL CAS, 0.05% SDS, or 2.5 μg/mL TM. The phosphorylated HpMpk1p was detected using the anti-phospho-p44/42 MAPK antibody, and the protein loading was monitored using an anti-β-actin antibody. The relative values of HpMpk1p phosphorylation to the basal level value of the wild-type were indicated, which were obtained by measuring the signal intensity of western blots with Quantity One 4.6.6 software (Bio-Rad). Error bars represent standard deviation of duplicate measurements. (C) Analysis of HpHog1 MAP kinase activation. The total soluble protein samples were obtained from the wild-type (WT), *Hppmt1*∆ (*∆1*), and *Hppmt4∆* (*∆4*) mutant strains, which were grown to early exponential phase in YPD containing 0.5 M NaCl for 2 hr. Immunoblotting was conducted using anti-phospho-p38 antibody to detect phospho-Hog1p and with anti-Hog1 antibody as a loading control. The strains used were wild-type 1BQ-LA (WT) and the *Hppmt1*∆ (*∆1*) and *Hppmt4*∆ (*∆4*) mutants.

In yeast, cell wall damage leads to the induction of the cell wall integrity (CWI) pathway, mediated by the mitogen-activated protein kinase (MAPK) Mpk1p, which results in numerous cellular responses [52]. To investigate whether the CWI pathway is activated due to cell wall damage caused by defective *O*-mannosylation in the *Hppmt4*∆ mutant, we analyzed the phosphorylation of HpMpk1p in the wild-type, *Hppmt1*∆, and *Hppmt4*∆ mutant strains by western blot analysis using the anti-phospho-p44/p42 antibody. Intriguingly, the basal level of phosphorylated HpMpk1p, a key kinase in the cell wall integrity pathway, was markedly reduced in the *Hppmt4*∆ mutant strain, while the phosphorylation of HpMpk1p was slightly induced in the *Hppmt1*∆ mutant compared to that in the wild-type strain in the absence of other external stresses (Fig 3A and 3B). However, despite the decrease in the basal level of phosphorylated HpMpk1p in the *Hppmt4*Δ strain, the phosphorylation level was increased upon treatment with various cell wall disturbing agents, just as in the wild-type strain. Compared to moderate activation of HpMpk1p found in response to CFW, CR, CAS and SDS, the phosphorylation of HpMpk1p increased more significantly in the presence of caffeine (Fig 3A and S3 Table). Particularly, treatment with TM led to the most dramatic activation of HpMpk1p phosphorylation among tested cell wall stressors (Fig 3B and S3 Table). These results suggest that the CWI signaling pathway can still be activated by cell wall damage, even when the *O*-mannosylation of surface sensors such as Wsc1p and Mid2p, normally catalyzed by the Pmt4 protein, is severely reduced.

A previous study in *S*. *cerevisiae* showed that the high osmolarity glycerol (HOG) pathway was activated in response to zymolyase, which lyses cell walls of viable yeast cells [53], suggesting cooperative cross-talk between the CWI and HOG signaling pathways [54,55]. We thus explored whether reduced *O*-mannosylation in *H*. *polymorpha* would also lead to stimulation of the HOG pathway, which is mostly dedicated to the adaptation of yeast cells to osmotic stress. Under normal conditions, the levels of phosphorylated HpHog1p were found to be slightly higher in the *Hppmt1*∆ and *Hppmt4*∆ mutant strains than in the wild-type (Fig 3C), implying that the defect in cell wall integrity in these *pmt* mutants activates the HOG pathway in *H*. *polymorpha* even under normal growth conditions. The wild-type, *Hppmt1*∆, and *Hppmt4*∆ mutant strains all showed further activation of the HOG pathway upon NaCl treatment. Altogether, these results indicate that, although the loss of HpPmt4p causes severe defects in *O*-mannosylation of cell surface sensor proteins, the mutant strain still retains its ability to activate the CWI and HOG MAPK signaling pathways in response to cell wall stress conditions.

### Analysis of a conditional *Hppmt1pmt4∆* double mutant

**Fig 4:**
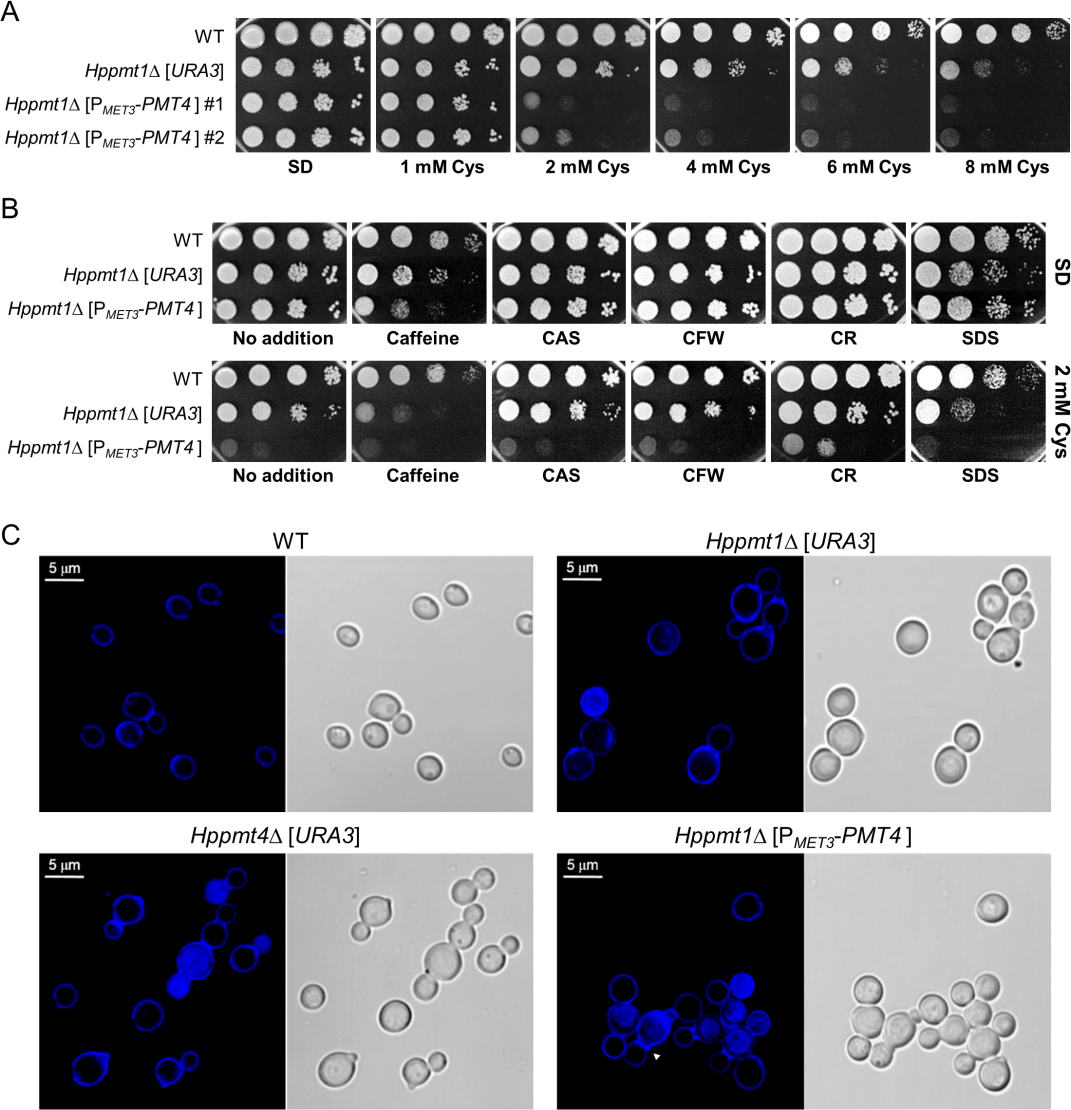
Morphological and growth phenotypes of the conditional *Hppmt1pmt4*∆ mutant strain. (A) Yeast cells were grown overnight in YPD and then adjusted to an OD_600_ of 1.0, diluted in 10-fold increments, and then each dilution was spotted onto SD plates supplemented with varying concentrations of cysteine. The pictures of the plates were taken after 5 days of incubation at 37°C. (B) A series of 10-fold dilutions of *H*. *polymorpha* strains was spotted on SD plates only or SD plates containing 10 mM caffeine, 0.3 μg/mL CAS, 0.1 mg/mL CFW, 3 mg/mL CR, or 0.005% SDS without or with 2 mM cysteine. Cells were incubated for 4 days. (C) Confocal micrographs of indicated strains (wild-type, *Hppmt1*∆, *Hppmt4*∆, and *Hppmt1pmt4*∆ mutants) grown for 1 day in SD liquid media containing 2 mM cysteine. Cells were fixed for 10 min in 3.7% formaldehyde and subsequently stained with the chitin-staining dye CFW.

Our previous study showed that the *Hppmt1pmt5*∆, *Hppmt1pmt6*∆, *Hppmt5pmt6*∆ double mutants, and even a *Hppmt1pmt5pmt6*∆ triple mutant, were viable [41]. To investigate the effect of *HpPMT4* deletion in the absence of HpPmt1p, we tried to construct an *Hppmt1pmt4∆* double mutant strain. Despite several trials of transformation of a *H*. *polymorpha pmt4* mutant strain with a *PMT1* disruption cassette, we could not obtain the *Hppmt1pmt4∆* double mutant strain. Along with the observation that *Hppmt4∆* mutant showed an extremely severe sensitivity to the Pmt1p inhibitor R3A-1c (Fig 1A), this implied that the simultaneous loss of *PMT1* and *PMT4* functions is most likely lethal to cells in *H*. *polymorpha*. To verify this hypothesis, the conditional *PMT4* allele under control of the *HpMET3* promoter was introduced in the *Hppmt1∆* mutant strain. This was accompanied with inactivation of the chromosomal wild-type *PMT4* allele. Expression of the *PMT4* conditional allele could be down-regulated by the controllable *HpMET3* promoter, whose expression is repressed by the supplementation of cysteine but is induced under sulfur-limited conditions [56]. The growth of the *Hppmt1*Δ [P_*MET3*_-*PMT4*] mutant strain was severely retarded on SD medium containing more than 2 mM cysteine, compared to that of the *Hppmt1∆* single mutant (Fig 4A and 4B). These results supported the theory that the concerted action of HpPmt1p and HpPmt4p is indispensable for growth of *H*. *polymorpha*.

We further investigated cell morphology of the *Hppmt* mutants through microscopic observation. As shown in Fig 4C, the *Hppmt1∆* and *Hppmt4∆* mutants showed an aberrant swollen or enlarged shape and formed chains of cells, whereas the wild-type cells exhibited normal growth as single yeast cells with simple buds. Moreover, the conditional *Hppmt1pmt4∆* double mutant displayed not only a large and dysmorphic phenotype but also prominent cell aggregation with an apparently aberrant (or random) budding pattern, reflecting inability of the cells to separate normally. To investigate the presence of chitin, the cells were stained with CFW. The birth scar, in which chitin has been degraded [57], was clearly observed in single cells of the wild-type strain. While the *Hppmt1∆* and *Hppmt4∆* single mutants showed a chitin deposition pattern similar to that of the wild-type cells, the conditional *Hppmt1pmt4∆* double mutant cells displayed unusual accumulation of chitin between connected cells (Fig 4C, arrow), which might result in a failure of daughter cells to dissociate properly from mother cells.

### Homo- and heterodimeric complex formation of HpPmt4 proteins

**Fig 5:**
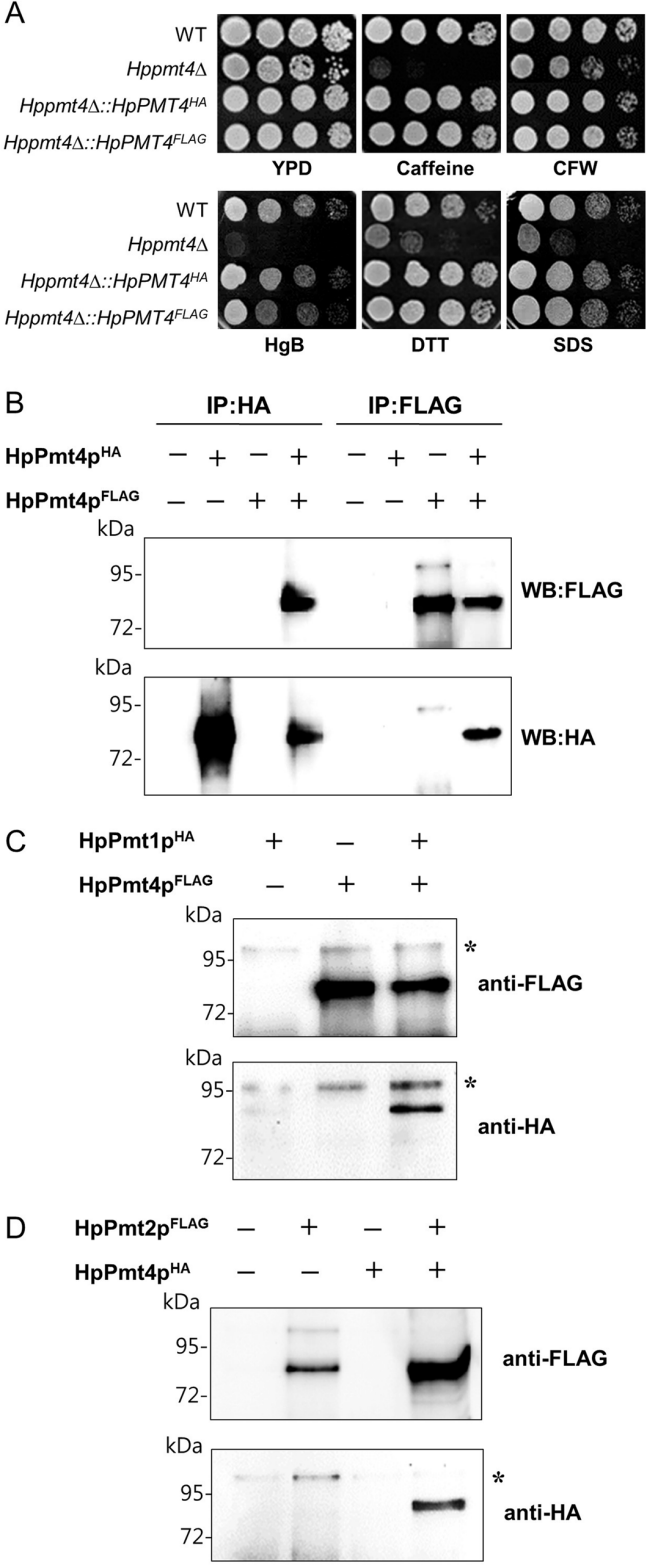
Complex formation of HpPmt4 proteins. (A) *In vivo* complementation analysis of epitope-tagged HpPmt4 proteins. A series of 10-fold dilutions of yeast strain expressing the epitope-tagged HpPmt4 proteins were spotted on YPD plates containing 10 mM caffeine, 0.1 mg/mL CFW, 5 μg/mL HgB, 0.01% SDS, 0.5 μg/mL TM, or 10 mM DTT and incubated for 2 days at 37°C. (B) Co-immunoprecipitation (co-IP) experiments to analyze interactions between HpPmt4 proteins. HpPmt4p^HA^ and HpPmt4p^FLAG^ were expressed individually or co-expressed in pairs in the *H*. *polymorpha* wild-type strain. SDC extracts were prepared and IP was performed using anti-HA agarose or anti-FLAG M2 affinity gel. Precipitates were resolved by 8% SDS-PAGE and analyzed by western blotting. Blots were sequentially probed with anti-HA and anti-FLAG antibodies. (C-D) Co-IP experiments to analyze interactions between HpPmt4p and either HpPmt1p (C) or HpPmt2p (D). HpPmt1p^HA^ and HpPmt4p^FLAG^ or HpPmt2p^FLAG^ and HpPmt4p^HA^ were expressed individually or co-expressed in pairs in the *H*. *polymorpha* wild-type strain. SDC extracts were prepared and co-IP was performed using anti-FLAG M2 affinity gel. Precipitates were analyzed by western blotting with anti-Flag or anti-HA antibody. The asterisk indicates a nonspecific protein band.

In *S*. *cerevisiae*, Pmt4p forms homomeric complexes *in vivo* [3]. To investigate whether this complex formation also occurs in *H*. *polymorpha*, we performed co-immunoprecipitation experiments using an HA epitope-tagged version of HpPmt4p (HpPmt4p^HA^) and FLAG epitope-tagged HpPmt4p (HpPmt4p^FLAG^). In order to confirm whether the epitope-tagged HpPmt4 proteins are functional *in vivo*, we tested functional complementation of the chemical reagent-sensitivity phenotype of the *Hppmt4*∆ mutant by expression of epitope-tagged HpPmt4 proteins. The *Hppmt4*∆ mutant cell was transformed with a plasmid containing HA- or FLAG-tagged HpPmt4p. As shown in Fig 5A, the expression of HA- or FLAG-tagged HpPmt4p in the *Hppmt4*∆ mutant complemented the sensitivity to several cell wall destabilizers and ER stressors. These complementation experiments demonstrate that C-terminal epitope-tagging does not inhibit the function of Pmt4 protein *in vivo* in *H*. *polymorpha*. Next, to detect Pmt4p-Pmt4p complexes, HpPmt4p^HA^ and HpPmt4p^FLAG^ proteins were co-expressed in the *Hppmt4*∆ mutant strain. Proteins solubilized from the crude membrane fractions by treatment with Triton X-100 and sodium deoxycholate were analyzed by co-immunoprecipitation experiments using anti-HA agarose or anti-FLAG M2 affinity gel. The immunoprecipitate obtained was resolved with 8% SDS-PAGE and detected by western blotting using antibodies against HA or FLAG. The results from the experiment with the *Hppmt4*∆ mutant strain co-expressing HpPmt4p^HA^ and HpPmt4p^FLAG^ demonstrated that HpPmt4p forms homomeric complexes (Fig 5B).

Unlike *S*. *cerevisiae* PMT members, PMT2 and PMT4 subfamily members in mammals and flies form a heteromeric complex [58,59]. Furthermore, a very recent study showed that *A*. *nidulans* PmtC (subfamily PMT4) forms not only homodimers but also heteromeric complexes with either PmtA (subfamily 2) or PmtB (subfamily 1) [21]. To investigate whether HpPmt4p can interact with either HpPmt1p or HpPmt2p in *H*. *polymorpha*, we constructed *H*. *polymorpha* strains co-expressing either an HA epitope-tagged version of HpPmt1p (HpPmt1p^HA^) and HpPmt4p^FLAG^, or FLAG epitope-tagged HpPmt2p (HpPmt2p^FLAG^) and HpPmt4p^HA^. Co-immunoprecipitations were performed with anti-FLAG M2 affinity gel by using the solubilized membrane proteins derived from both strains, and then the corresponding immunoprecipitates were probed with anti-FLAG and anti-HA antibodies, respectively. When probed with anti-FLAG antibody, two protein bands of approximately 86 kDa and 89 kDa were detected in the strains bearing HpPmt4p^FLAG^ (Fig 5C, upper panel) and HpPmt2p^FLAG^ (Fig 5D, upper panel), respectively. Immunoblots probed with anti-HA antibody displayed the bands of HpPmt1p^HA^ (~91 kDa) and HpPmt4p^HA^ (~86 kDa) in the samples from the HpPmt1p^HA^ and HpPmt4p^FLAG^ co-expressing strain (Fig 5C, lower panel) and HpPmt2p^FLAG^ and HpPmt4p^HA^ co-expressing strain (Fig 5D, lower panel), respectively. Furthermore, the HpPmt1p-HpPmt4p and HpPmt2p-HpPmt4p heterodimer complexes were also observed in the *H*. *polymorpha* DL-1 strains (S3 Fig), although their band intensities were much lower than the band intensity detected in the strain harboring HpPmt1p^HA^ and HpPmt2p^FLAG^. These results strongly suggest that *H*. *polymorpha* Pmt4 proteins also participate in the formation of heteromeric complexes with either HpPmt1p or HpPmt2p.

## Discussion

Protein *O*-mannosylation, initiated by PMT family members in the ER, is known to play important roles in various biological processes, including cell morphology, protein stability [27,60], protein secretion [2,25], cell wall integrity [5], the budding process [24], sorting [61] and localization of proteins, and virulence of pathogens [9,62]. Unlike the PMT1/PMT2 subfamilies, which are highly redundant, the PMT4 subfamily has only one representative per species. In the present study, we report the functional and molecular features of HpPmt4p, a unique representative of the PMT4 subfamily in a thermotolernat methylotrophic yeast *H*. *polymorpha* with high industrial potential. We showed that the function of HpPmt4p, as a key player in *O*-mannosylation of cell surface proteins, is critical for resistance to cell wall and osmotic stresses. Moreover, we presented a novel finding that HpPmt4p forms not only a homomeric complex but also a heteromeric complex with either HpPmt1p or HpPmt2p in *H*. *polymorpha*, which is distinctive from *S*. *cerevisiae* that is known to form exclusively heterocomplex between the PMT1 and PMT2 subfamilies.

The physiological effect of *PMT4* deletion has been investigated in several organisms from yeast to humans. In *S*. *cerevisiae*, disruption of *PMT4* exhibited no obvious growth defect phenotype, and only multiple *PMT* gene disruptions including a *pmt4* mutation partially showed conspicuous phenotypes [5]. For example, the *S*. *cerevisiae pmtlpmt3pmt4*Δ triple mutant was only slightly affected in spore germination, whereas the *S*. *cerevisiae pmt2pmt4*Δ double mutant can grow only when osmotically stabilized. The *S*. *cerevisiae pmt1pmt4*Δ and *pmt1pmt3pmt4*Δ mutants are temperature sensitive, whereas *S*. *cerevisiae pmtlpmt2pmt4*Δ and *pmt2pmt3pmt4*Δ mutations seemed to be lethal. In contrast to *S*. *cerevisiae PMT4*, just a single deletion of *PMT4* homologs of other yeast and filamentous fungal species generated apparent defects in cell morphogenesis. In *S*. *pombe*, the *oma4* single mutant strain, which is a deletion of the *ScPMT4* homolog, forms an abnormal cell wall and septum, thereby exhibiting severely altered cell morphology and cell-cell separation [63]. Hyphal formation of a *C*. *albicans pmt4* mutant was defective on defined Lee’s medium but increased under embedded or hypoxic conditions [9]. In the opportunistic human pathogen *C*. *neoformans*, *CnPMT4* is also crucial for cell morphogenesis and virulence [10,62]. In another opportunistic human pathogenic fungus, *A*. *fumigatus*, deletion of *AfPMT4* not only affects cell growth, hyphal morphology, and conidiation but also causes hypersensitivity to echinocandins. In the plant pathogen *U*. *maydis*, *UmPMT4* is required for fungal pathogenesis through its essential role in appressorium formation and plant penetration [15]. Similarly, in another plant pathogenic fungus, *B*. *cinerea*, *PMT4* plays a specific role in virulence [8]. Furthermore, mutations of the *D*. *melanogaster POMT1* (PMT4 subfamily) causes defects in muscle attachment and changes in muscle contraction during larval development [64]. In mice, targeted deletion of the *POMT1* gene results in embryonic lethality, which is due to structural and functional defects in the first basal membrane of the embryo [65]. Mutations in the human *POMT1* cause not only Walker-Warburg syndrome (WWS) [66] but also muscle-eye-brain disease (MEB) [67]. In the present study, we also showed that just a single deletion of *HpPMT4* resulted in severe defects in cell wall integrity and stress resistance of *H*. *polymorpha*, indicating certain unique roles of HpPmt4p in *O*-mannosylation of glycoproteins, which cannot be fully complemented by the function of other PMT members.

Previous studies in *S*. *cerevisiae* and *C*. *albicans* showed the transcriptional alteration of *PMT* genes in response to defective *O*-mannosylation [52, 68]. To explore the transcriptional regulation of the *H*. *polymorpha PMT* genes, we determined the mRNA levels of all *PMT* subfamily genes in the wild-type and *pmt4∆* mutant strains. In contrast to the increased levels of the *PMT1* subfamily (*PMT1* and *PMT5*) transcripts in the *pmt4* mutant of *C*. *albicans* [68], any compensatory up-regulation of other *PMT* genes was not detected in the *H*. *polymorpha pmt4*∆ mutant (S4A Fig). In addition, the transcript levels of all *H*. *polymorpha PMT* subfamilies were not significantly changed even in the presence of TM or a Pmt1p inhibitor. On the other hand, all five *PMT* genes of *P*. *pastoris* were reported to be extremely down-regulated during growth on methanol as compared to that on glycerol due to lower energy availability [11]. Thus, we tested the expression levels of *H*. *polymorpha PMT* genes on different culture media such as sulfur-free B media (synthetic media with 2% glucose without any sulfur source) [69], SD medium (synthetic minimal medium), and YPM medium containing 2% methanol as the sole carbon source (S4B Fig). The qRT-PCR analysis showed that although there was no significant alteration in the expression levels of the *HpPMT* genes in B and SD media, the *PMT1*, *PMT2*, and *PMT6* genes were dramatically repressed by more than 2-fold on methanol-containing media. It is notable that, among the *HpPMT* genes coding for three major isoforms Pmt1p, Pmt2p, and Pmt4p, the transcript level of *HpPMT4* was found to be maintained at a relatively constant level under various culture conditions tested in this study.

In yeast, damage to the cell wall is sensed by plasma membrane sensors in the WSC family (Wsc1p to Wsc4p) and Mid2p, which are highly *O*-mannosylated surface proteins [27]. We demonstrate that HpPmt4p mediates *O*-mannosylation primarily of cell surface proteins, such as HpWsc1 and HpMid2 proteins, which are involved in cell wall integrity signaling. Previously, we reported that the basal level of Mpk1 MAPK phosphorylation highly increased in the *Hppmt1pmt5∆* and *Hppmt1pmt6∆* mutant strains, in which hypomannosylation was also observed for HpWsc1^HA^ and HpMid2^FLAG^ proteins [41]. However, in the present study, we observed that the basal level of the phosphorylated Mpk1p in the *Hppmt4*∆ mutant strain was dramatically lower than that in the wild-type strain under normal conditions. This suggests that HpWsc1p and HpMid2p might be mannosylated by both PMT1/2 family members and Pmt4p, but in distinct regions, as shown in *S*. *cerevisiae* [27], and that the *O*-mannosylation of these sensor proteins by Pmt4p is required for maintaining basal activity of cell wall sensors under normal cell conditions.

**Fig 6:**
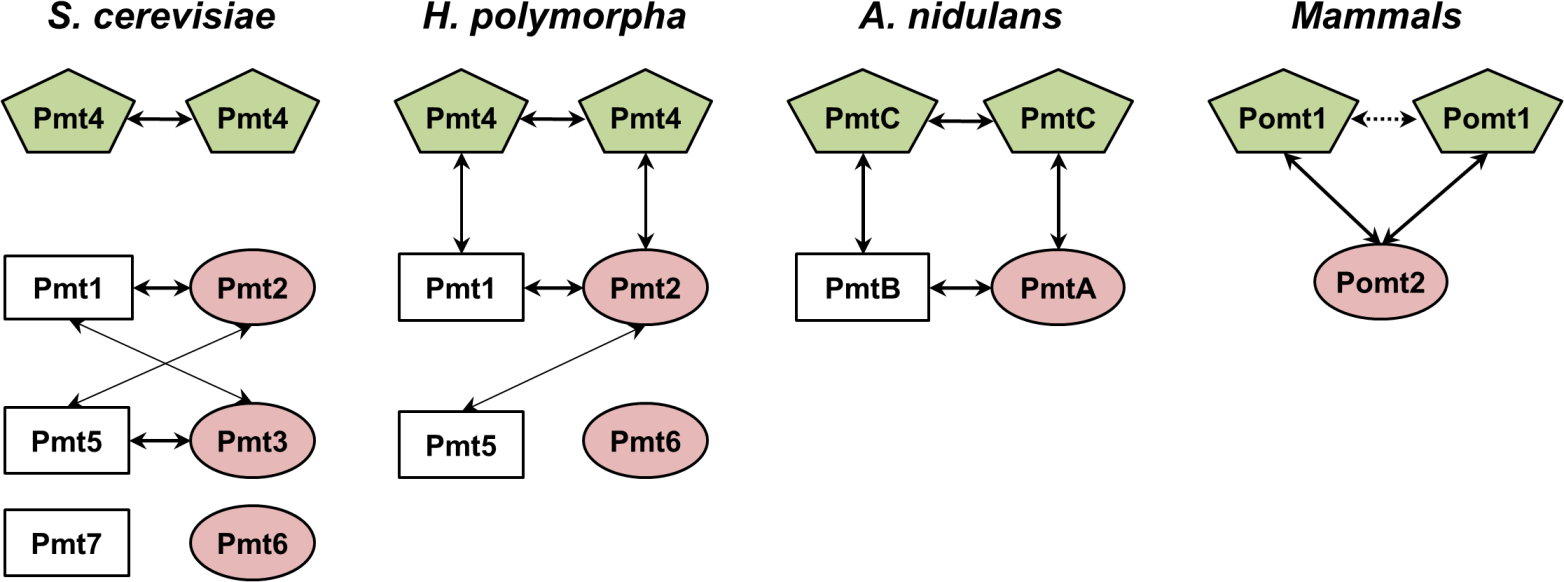
Proposed evolution of the *PMT* gene family and their complex formation. In *S*. *cerevisiae*, the PMT1 subfamily forms heteromeric complexes with the PMT2 subfamily while the PMT4 subfamily forms homomeric complexes. Pmt1p interacts mainly with Pmt2p, while interaction between Pmt1p and Pmt3p, or interaction of Pmt2p with Pmt5p occurs only in the absence of their preferred partners. In *A*. *nidulans*, PmtB (subfamily PMT1), PmtA (subfamily PMT2), and PmtC (subfamily PMT4) form heteromeric complexes in every possible subfamily combination, and PmtC also interacts with itself. In mammals, Pomt2 (subfamily PMT2) forms a heteromeric complex with Pomt1 (subfamily PMT4). In *H*. *polymorpha*, Pmt1p forms heteromeric complexes mainly with Pmt2p. Interaction between HpPmt2p and HpPmt5p can occur in the absence of HpPmt1p. HpPmt4p forms both homomeric complexes, and heteromeric complexes with either HpPmt1p or HpPmt2p. Rectangles indicate the PMT1 subfamily, ovals indicate the PMT2 subfamily, and pentagons indicate the PMT4 subfamily.

Until now, it has generally been considered that a member of the PMT1 subfamily interacts with a member of the PMT2 subfamily, whereas the PMT4 subfamily member self-dimerizes for maximal *O*-mannosyltransferase activity in yeasts and other fungi. However, in contrast to the findings for fungal species, the PMT2 subfamily and PMT4 subfamily are reported to form a heterodimeric complex in mammals. The co-immunoprecipitation experiments in *S*. *cerevisiae* showed that Pmt1p-Pmt3p and Pmt2p-Pmt5p are present in minor amounts, although Pmt1p-Pmt2p and Pmt5p-Pmt3p were the predominant complexes [3]. Similarly, in *H*. *polymorpha*, Pmt2p can also interact with Pmt5p, a minor member of the PMT1 family, in the absence of the preferred partner Pmt1p [41]. Both of these yeasts have a redundant member of the PMT1 and PMT2 subfamilies, which could substitute if the dominant members are lost. Notably, a very interesting report on complex formation in all pairwise combinations of three *A*. *nidulans* Pmts, one from each subfamily, was recently presented [21], describing the possibility of complex formation between PMT1 or PMT2 subfamily and PMT4 subfamily members in other fungal species. Our data from the co-immunoprecipitation analysis of the *H*. *polymorpha* wild-type strains co-expressing HpPmt1p^HA^ and HpPmt4p^FLAG^ or HpPmt2p^FLAG^ and HpPmt4p^HA^ strongly support that Pmt1p-Pmt4p and Pmt2p-Pmt4p complexes are also present in *H*. *polymorpha*. To our knowledge, this is the first case where the formation of Pmt1p-Pmt4p and Pmt2p-Pmt4p complexes is shown to occur in a yeast species that possesses a redundant member of the PMT1 and PMT2 subfamilies. Compared to the Pmt proteins of the budding yeasts *S*. *cerevisiae* and *H*. *polymorpha*, which contain two to three redundant members, many filamentous fungi, including *A*. *nidulans*, possess only one representative from each PMT subfamily. Animals have only the PMT2 and PMT4 subfamilies, except that all Pmts are missing in the nematode *Caenorhabditis elegans* [3]. Based on these findings, it can be speculated that *PMT* gene families have most likely evolved to lose redundant genes, while the complex formation necessary for enzymatic activity has diversified with simplification of the redundant *PMT* genes (Fig 6).

In this study, we showed that the function of *H*. *polymorpha* Pmt4 protein is critical, particularly for *O*-mannosylation of membrane proteins, which is crucial for protein stability. Likewise, in *S*. *cerevisiae*, the integral transmembrane proteins Axl2/Bud10 require Pmt4p-mediated *O*-mannosylation for their proper folding and the avoidance of degradation in the Golgi apparatus [24]. In addition, un-*O*-mannosylated Fus1p accumulates in late Golgi structures, suggesting that *O*-mannosyl modification by Pmt4p functions as a sorting determinant for cell surface delivery of Fus1p [42]. These results point to different roles of Pmt4p from those of PMT1/2 subfamily members in *O*-mannosylation of glycoproteins. It seems that PMT1/2 subfamily members are mainly responsible for *O*-mannosylation of secretory proteins, while Pmt4p is instead focused on surface membrane proteins. Along with *N*-glycosylation engineering, *O*-glycoengineering has been attempted in order to produce recombinant secretory proteins with mammalian-type glycosylation patterns in several yeast species, including *S*. *cerevisiae* [70,71], *P*. *pastoris* [72], and *Ogataea minuta* [73]. Thus, understanding the specific functions of each PMT member in protein *O*-mannosylation in yeast is important to elucidate their differential effects on different target proteins, which should be considered when developing yeast expression systems either for production of secretory glycoproteins or for surface display of membrane glycoproteins.

## Supporting Information

S1 Fig Effect of glycerol and sucrose on growth phenotypes of *H*. *polymorpha pmt* mutant strains. *H*. *polymorpha* cells were cultivated on YPD only plates (A) or YPD plates containing 1 M glycerol (B) or 1 M sucrose (C). The overnight cultivated wild-type 1BQ-LA and *Hppmt* mutant cells were adjusted to an OD_600_ of 1.0, diluted by 10-fold serial dilutions down to a 10^−4^ dilution, and then spotted onto YPD plates supplemented with various stress reagents at indicated concentrations. Plates were incubated for 2 days.
(DOCX)

S2 Fig Complementation of *O*-mannosylation defects in the *Hppmt4*∆ mutant strains by reintroduction of functional *HpPMT4*. Cell extracts from the *H*. *polymorpha* wild-type (lane 1), *Hppmt1*∆ (lane 2), *Hppmt4*∆ (lane 3) and *Hppmt4Δ*::*HpPMT4* (lane 4) expressing either HpWsc1p^HA^ (A) or HpMid2p^FLAG^ (B). HpWsc1p^HA^ were subjected to 8% SDS-PAGE followed by western blot analysis with anti-HA antibody. HpMid2p^FLAG^ was resolved by 6% SDS-PAGE (left panel) and 12% SDS-PAGE (right panel). Blots were sequentially probed with anti-FLAG antibody. The β-actin protein indicates equal loading of the lanes.
(DOCX)

S3 Fig Complex formation of HpPmt4 proteins in *H*. *polymorpha* DL-1 strains. Co-immunoprecipitation experiments to analyze interactions between HpPmt4p and either HpPmt1p or HpPmt2p in the *H*. *polymorpha* DL-1 strain background. HpPmt1p^HA^ and HpPmt4p^FLAG^, or HpPmt2p^FLAG^ and HpPmt4p^HA^, were expressed individually or co-expressed in pairs in the wild-type strain. SDC extracts were prepared and IP was performed using anti-FLAG M2 affinity gels. Precipitates were analyzed by western blotting with anti-Flag (upper panel) or anti-HA (lower panel) antibodies. The asterisk indicates a non-specific protein band.
(DOCX)

S4 Fig Relative mRNA levels of the *HpPMT* genes under various culture conditions. Total RNA of three independent cultures was isolated according to the hot phenol extraction method [74] from the indicated strains. Relative expression levels of the five *HpPMT* genes (*HpPMT1*, *HpPMT2*, *HpPMT4*, *HpPMT5*, and *HpPMT6*) were determined by quantitative real-time PCR using SYBR Premix Ex Taq II (TAKARA) and 10 pmol of each forward and reverse oligonucleotide primer (S2 Table). Transcript levels relative to the *ACT1* transcript levels were calculated by the modified formula 2^mean Ct *ACT1*^/2^mean Ct target^. Error bars represent standard deviation of triplicated data. (A) The relative levels of five *HpPMT* transcripts under several stress conditions. *H*. *polymorpha* cells were grown to an OD_600_ of 1.0 in YPD medium at 37°C, and then treated with 2.5 μg/mL TM or 60 μM Pmt1p inhibitor (R3A-1c) for 2 hr. (B) The relative levels of *PMT* mRNA in various media. The *H*. *polymorpha* wild-type cells cultured overnight were inoculated at an initial OD_600_ of 0.4, grown to mid-logarithmic phase (OD_600_ of 1.0) in YPD, and then transferred into YPM medium containing 2% methanol or B or SD minimal medium and were further cultivated for 2 hr.
(DOCX)

S1 Table List of plasmids used in this study. (DOCX)

S2 Table List of primer sequences used in this study. (DOCX)

S3 Table Relative fold change of HpMpk1p phosphorylation after treatment with cell wall stressors. (DOCX)
