# Supplementary material for: Hansenula polymorpha Pmt4p Plays Critical Roles in O-Mannosylation of Surface Membrane Proteins and Participates in Heteromeric Complex Formation
Source: PLoS One. 2015 Jul 2;10(7):e0129914. doi: 10.1371/journal.pone.0129914 (PMC4489896; doi:10.1371/journal.pone.0129914)
Supplement: S1 Fig — H. polymorpha cells were cultivated on YPD only plates (A) or YPD plates containing 1 M glycerol (B) or 1 M sucrose (C). The overnight cultivated wild-type 1BQ-LA and Hppmt mutant cells were adjusted to an OD600 of 1.0, diluted by 10-fold serial dilutions down to a 10−4 dilution, and then spotted onto YPD plates supplemented with various stress reagents at indicated concentrations. Plates were incubated for 2 days. (DOCX) [file pone.0129914.s001.docx]

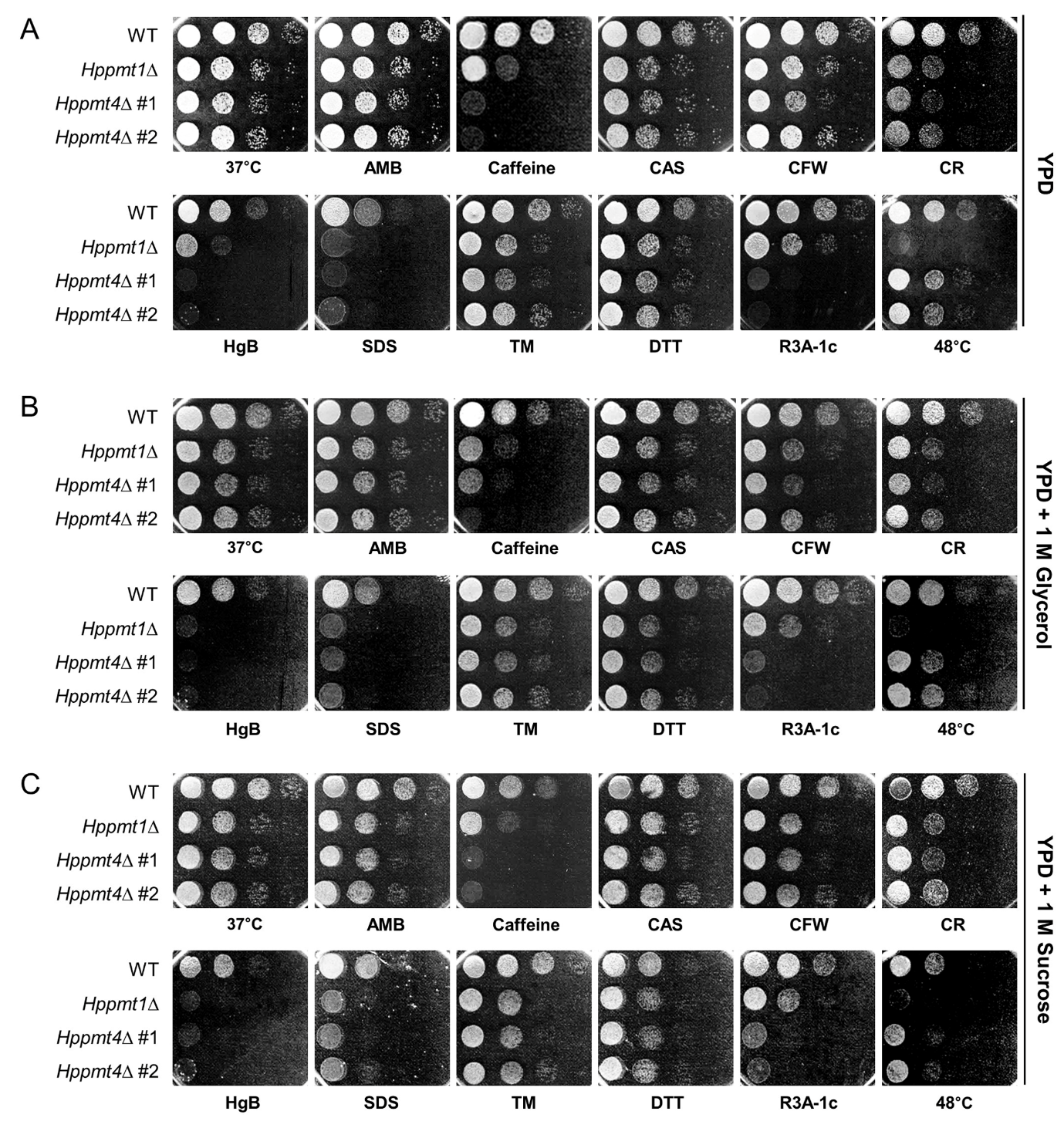


**S1 Figure. Effect of glycerol and sucrose on growth phenotypes of *H. polymorpha* *pmt* mutant strains.** *H. polymorpha* cells were cultivated on YPD only plates (A) or YPD plates containing 1 M glycerol (B) or 1 M sucrose (C). The overnight cultivated wild-type 1BQ-LA and *Hppmt* mutant cells were adjusted to an OD_600_ of 1.0, diluted by 10-fold serial dilutions down to a 10^-4^ dilution, and then spotted onto YPD plates supplemented with various stress reagents at indicated concentrations. Plates were incubated for 2 days.
