# Supplementary material for: Hansenula polymorpha Pmt4p Plays Critical Roles in O-Mannosylation of Surface Membrane Proteins and Participates in Heteromeric Complex Formation
Source: PLoS One. 2015 Jul 2;10(7):e0129914. doi: 10.1371/journal.pone.0129914 (PMC4489896; doi:10.1371/journal.pone.0129914)
Supplement: S2 Fig — Cell extracts from the H. polymorpha wild-type (lane 1), Hppmt1∆ (lane 2), Hppmt4∆ (lane 3) and Hppmt4Δ::HpPMT4 (lane 4) expressing either HpWsc1pHA (A) or HpMid2pFLAG (B). HpWsc1pHA were subjected to 8% SDS-PAGE followed by western blot analysis with anti-HA antibody. HpMid2pFLAG was resolved by 6% SDS-PAGE (left panel) and 12% SDS-PAGE (right panel). Blots were sequentially probed with anti-FLAG antibody. The β-actin protein indicates equal loading of the lanes. (DOCX) [file pone.0129914.s002.docx]

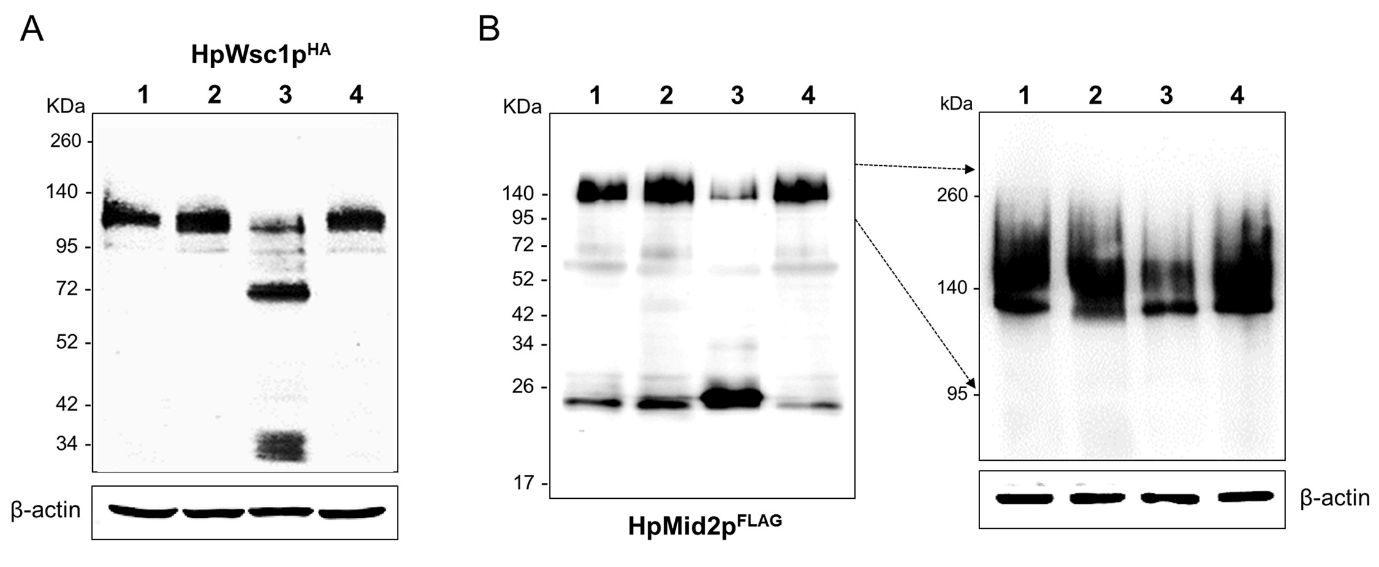


**S2 Figure. Complementation of *O*-mannosylation defects in the *Hppmt4*∆ mutant strains by reintroduction of functional *HpPMT4*.** Cell extracts from the *H. polymorpha* wild-type (lane 1), *Hppmt1*∆ (lane 2), *Hppmt4*∆ (lane 3) and *Hppmt4Δ::HpPMT4* (lane 4) expressing either HpWsc1p^HA^ (A) or HpMid2p^FLAG^ (B). HpWsc1p^HA^ were subjected to 8% SDS-PAGE followed by western blot analysis with anti-HA antibody. HpMid2p^FLAG^ was resolved by 6% SDS-PAGE (left panel) and 12% SDS-PAGE (right panel). Blots were sequentially probed with anti-FLAG antibody. The β-actin protein indicates equal loading of the lanes.
