# Supplementary material for: Hansenula polymorpha Pmt4p Plays Critical Roles in O-Mannosylation of Surface Membrane Proteins and Participates in Heteromeric Complex Formation
Source: PLoS One. 2015 Jul 2;10(7):e0129914. doi: 10.1371/journal.pone.0129914 (PMC4489896; doi:10.1371/journal.pone.0129914)
Supplement: S3 Fig — Co-immunoprecipitation experiments to analyze interactions between HpPmt4p and either HpPmt1p or HpPmt2p in the H. polymorpha DL-1 strain background. HpPmt1pHA and HpPmt4pFLAG, or HpPmt2pFLAG and HpPmt4pHA, were expressed individually or co-expressed in pairs in the wild-type strain. SDC extracts were prepared and IP was performed using anti-FLAG M2 affinity gels. Precipitates were analyzed by western blotting with anti-Flag (upper panel) or anti-HA (lower panel) antibodies. The asterisk indicates a non-specific protein band. (DOCX) [file pone.0129914.s003.docx]

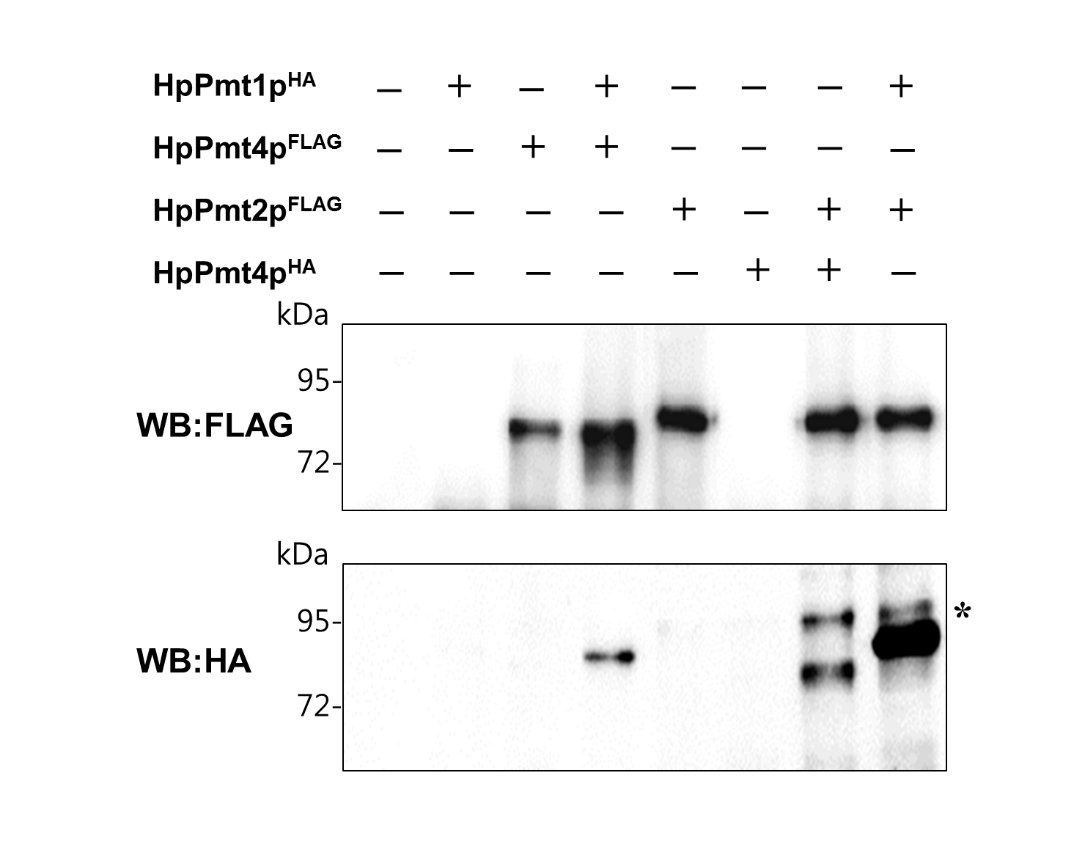


**S3 Figure. Complex formation of HpPmt4 proteins in *H. polymorpha* DL-1 strains.** Co-immunoprecipitation experiments to analyze interactions between HpPmt4p and either HpPmt1p or HpPmt2p in the *H. polymorpha* DL-1 strain background. HpPmt1p^HA^ and HpPmt4p^FLAG^, or HpPmt2p^FLAG^ and HpPmt4p^HA^, were expressed individually or co-expressed in pairs in the wild-type strain. SDC extracts were prepared and IP was performed using anti-FLAG M2 affinity gels. Precipitates were analyzed by western blotting with anti-Flag (upper panel) or anti-HA (lower panel) antibodies. The asterisk indicates a non-specific protein band.
