# Supplementary material for: Hansenula polymorpha Pmt4p Plays Critical Roles in O-Mannosylation of Surface Membrane Proteins and Participates in Heteromeric Complex Formation
Source: PLoS One. 2015 Jul 2;10(7):e0129914. doi: 10.1371/journal.pone.0129914 (PMC4489896; doi:10.1371/journal.pone.0129914)
Supplement: S4 Fig — Total RNA of three independent cultures was isolated according to the hot phenol extraction method [74] from the indicated strains. Relative expression levels of the five HpPMT genes (HpPMT1, HpPMT2, HpPMT4, HpPMT5, and HpPMT6) were determined by quantitative real-time PCR using SYBR Premix Ex Taq II (TAKARA) and 10 pmol of each forward and reverse oligonucleotide primer (S2 Table). Transcript levels relative to the ACT1 transcript levels were calculated by the modified formula 2mean Ct ACT1/2mean Ct target. Error bars represent standard deviation of triplicated data. (A) The relative levels of five HpPMT transcripts under several stress conditions. H. polymorpha cells were grown to an OD600 of 1.0 in YPD medium at 37°C, and then treated with 2.5 μg/mL TM or 60 μM Pmt1p inhibitor (R3A-1c) for 2 hr. (B) The relative levels of PMT mRNA in various media. The H. polymorpha wild-type cells cultured overnight were inoculated at an initial OD600 of 0.4, grown to mid-logarithmic phase (OD600 of 1.0) in YPD, and then transferred into YPM medium containing 2% methanol or B or SD minimal medium and were further cultivated for 2 hr. (DOCX) [file pone.0129914.s004.docx]

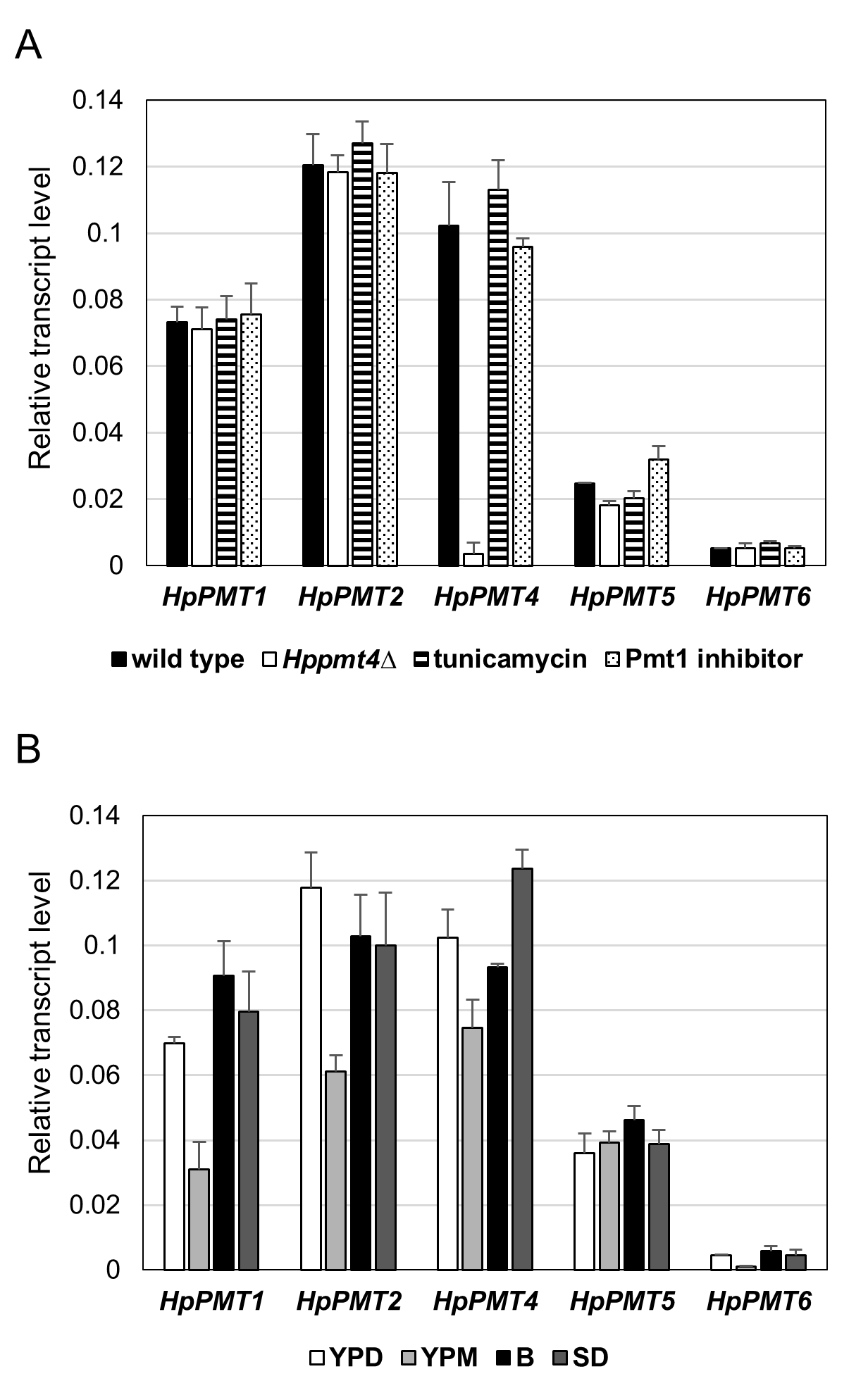


**S4 Figure. Relative mRNA levels of the *HpPMT* genes under various culture conditions.** Total RNA of three independent cultures was isolated according to the hot phenol extraction method [[74](#_ENREF_74)] from the indicated strains. Relative expression levels of the five *HpPMT* genes (*HpPMT1*, *HpPMT2*, *HpPMT4*, *HpPMT5*, and *HpPMT6*) were determined by quantitative real-time PCR using SYBR Premix Ex Taq II (TAKARA) and 10 pmol of each forward and reverse oligonucleotide primer (S2 Table). Transcript levels relative to the *ACT1* transcript levels were calculated by the modified formula 2^mean Ct^*^ACT1^*/2^mean Ct target^. Error bars represent standard deviation of triplicated data. (A) The relative levels of five *HpPMT* transcripts under several stress conditions. *H. polymorpha* cells were grown to an OD_600_ of 1.0 in YPD medium at 37°C, and then treated with 2.5 μg/mL TM or 60 µM Pmt1p inhibitor (R3A-1c) for 2 hr. (B) The relative levels of *PMT* mRNA in various media. The *H. polymorpha* wild-type cells cultured overnight were inoculated at an initial OD_600_ of 0.4, grown to mid-logarithmic phase (OD_600_ of 1.0) in YPD, and then transferred into YPM medium containing 2% methanol or B or SD minimal medium and were further cultivated for 2 hr.
