# Supplementary material for: Hansenula polymorpha Pmt4p Plays Critical Roles in O-Mannosylation of Surface Membrane Proteins and Participates in Heteromeric Complex Formation
Source: PLoS One. 2015 Jul 2;10(7):e0129914. doi: 10.1371/journal.pone.0129914 (PMC4489896; doi:10.1371/journal.pone.0129914)
Supplement: S1 Table — (DOCX) [file pone.0129914.s005.docx]

**S1 Table**. List of plasmids used in this study.

| **Plasmid** | **Description** | **Reference** |  |
| --- | --- | --- | --- |
| pTHpURA3-LZ | pGEM-T-based vector carrying the *lacZ-HpURA3-lacZ* pop-out cassette | [48] |  |
| pTHpLEU2-NS(c) | pGEM-T-based vector carrying the *HpLEU2* gene | [41] |  |
| pSS18 | pUC-based vector carrying *PMT1* disruption cassette with *LEU2* selection marker | [42] |  |
| pCSS27 | pBC-based vector carrying *PMT4* disruption cassette with *LEU2* selection marker and *ARS* | this study |  |
| pDUMOX-msdS (HA-HDEL) | pBC-based vector carrying the HA-HDEL-tagged *Aspergillus saitoi* alpha-1,2-mannosidase under the MOX promoter and *HpURA3* marker | [37] |  |
| pDUM3P600-PMT4D | pDUMOX-msdS carrying the C-terminal truncated *HpPMT4* under the *MET3* promoter | this study |  |
| pHIGAZ-6HA | pUC-based vector with a zeocin marker, the *HpGAP* promoter, and six copies of hemagglutinin (HA) tag | [41] |  |
| pHIGAHT-4FLAG | pUC-based vector with a hygromycin B marker, the *HpGAP* promoter, and four copies of FLAG tag | [41] |  |
| pHIGAZ-4FLAG | pUC-based vector with a zeocin marker, the *HpGAP* promoter, and four copies of FLAG tag | this study |  |
| pHINHT-HpPMT4F | pHIGAHT-4FLAG carrying the Flag-tagged *HpPMT4* with the native promoter | this study |  |
| pDUN-HpPMT4F | pDUMOX-msdS carrying HA-tagged *HpPMT4* with the native promoter | this study |  |
| pHINZ-HpPMT4H | pHIGAZ-6HA carrying the HA-tagged *HpPMT4* with the native promoter | this study |  |
| pHIGAZ-HpPMT1H | pHIGAZ-6HA carrying the HA-tagged *HpPMT1* under the *HpGAP* promoter | [41] |  |
| pHINZ-HpPMT1H | pHIGAZ-6HA carrying the HA-tagged *HpPMT1* with the native promoter | this study |  |
| pHIHT-HpPMT2F | pHIGAHT-4FLAG carrying the Flag-tagged *HpPMT2* gene | [41] |  |
| pHIGAZ-HpPMT2F | pHIGAHT-4FLAG carrying the Flag-tagged *HpPMT2* under the *HpGAP* promoter | [41] |  |
| pHINZ-HpWSC1H | pHIGAZ-6HA carrying the HA-tagged *HpWSC1* with the native promoter | this study | |
| pHINZ-HpMID2F | pHIGAZ-4FLAG carrying the FLAG-tagged *HpMID2* with the native promoter | this study |  |
| pMOX-YPS1ct-His | pBC-based vector carrying the C-terminal truncated HpYPS1 with C-terminal His tag under the MOX promoter and *HpLEU2* markers | [49] |  |
| pDLUMOX-HpYPS1H | pMOX-YPS1ct-His carrying the C-terminal truncated *HpYPS1* with C-terminal His tag and *HpURA3* markers | this study |  |
