# Supplementary material for: Hansenula polymorpha Pmt4p Plays Critical Roles in O-Mannosylation of Surface Membrane Proteins and Participates in Heteromeric Complex Formation
Source: PLoS One. 2015 Jul 2;10(7):e0129914. doi: 10.1371/journal.pone.0129914 (PMC4489896; doi:10.1371/journal.pone.0129914)
Supplement: S2 Table — (DOCX) [file pone.0129914.s006.docx]

**S2 Table**. List of primer sequences used in this study.

| **Primer name** | **Sequence description** | **Purpose** |
| --- | --- | --- |
| HpPMT4D_F | gtagcagacaatgccctcaa | Construction of *Hppmt4* mutant |
| HpPMT4D_R | gatttccaggagactcttgg | " |
| NheI-HpPMT4_F | taat**gctagc**tcgttgtctggaattggc | Construction of Flag-tagged *HpPMT4* |
| HpPMT4_R-AscI | t**ggcgcgcc**tttagcgaaatgcaatttga | " |
| SalI-HpMET3_F | tgag**gtcgac**tggccaccttgcacacaga | Construction of a conditional *Hppmt1pm4* mutant |
| HpMET3_R-EcoRI | gt**atcgat**gcattca**gaattc**cattttgggaggagagtcg | " |
| EcoRI-HpPMT4D_F | ca**gaattc**atggctcctaaacagagg | " |
| HpPMT4D-ClaI_R | ca**atcgat**ggcctttcttgatgtcca | " |
| BglII-HpWSC1_pm | cg**agatct**atcttggcaatctcgtcc | Construction of HA-tagged *HpWSC1* |
| HpWSC1_R-AscI | tat**ggcgcgccgg**attcttatcgtcgtcggg | " |
| BglII-HpMID2_pm | cg**agatct**tcgctctgaagtacgaca | Construction of FLAG-tagged *HpMID2* |
| HpMID2_R-AscI | tat**ggcgcgcc**aaaagtggtaccgggatt | " |
| BglII-HpPMT1_F | cg**agatct**gccagtgcaaagaaggac | Construction of Flag-tagged *HpPMT1* |
| HpPMT1_R-XhoI | attggcact**ctcgag**cac | " |
| HpPMT1F-RT | cgcagacgttggtatcggct | Detection of *HpPMT1* transcript |
| HpPMT1B-RT | cgtgatctgctgttgcccag | " |
| NCYCHpPMT2F-RT | caacgctttggtgccagacc | Detection of *HpPMT2* transcript |
| NCYCHpPMT2B-RT | atttcacgttgtcgtcgccc | " |
| HpPMT4F-RT | atcgctccctgaatcctggc | Detection of *HpPMT4* transcript |
| HpPMT4B-RT | agacccacttcgagccacca | " |
| HpPMT5F-RT | cagagatcggttcgaagcgg | Detection of *HpPMT5* transcript |
| HpPMT5B-RT | cgtcgtgtccttctgcccat | " |
| HpPMT6F-RT | ccaggtgacgacgtacgggt | Detection of *HpPMT6* transcript |
| HpPMT6B-RT | ttggtgagctcgtgctgcag | " |
| HpACT1F-RT | tccaggctgtgctgtcgttg | Detection of *HpACT1* transcript |
| HpACT1B-RT | ccggccaagtcgattctcaa | " |
|  |  |  |
| * Bold type indicates restriction enzyme sites. | |  |
