# Supplementary material for: Hansenula polymorpha Pmt4p Plays Critical Roles in O-Mannosylation of Surface Membrane Proteins and Participates in Heteromeric Complex Formation
Source: PLoS One. 2015 Jul 2;10(7):e0129914. doi: 10.1371/journal.pone.0129914 (PMC4489896; doi:10.1371/journal.pone.0129914)
Supplement: S3 Table — (DOCX) [file pone.0129914.s007.docx]

**S3 Table**. Relative fold change of HpMpk1p phosphorylation after treatment with cell wall stressors*.

|  | Caffeine | CFW | CR | CAS | SDS | TM |
| --- | --- | --- | --- | --- | --- | --- |
| WT | 2.1 | 1.4 | 1.3 | 1.4 | 1.6 | 3.5 |
| *Hppmt1*∆ | 2.0 | 1.3 | 1.4 | 1.7 | 1.9 | 3.1 |
| *Hppmt4*∆ | 2.2 | 1.2 | 1.8 | 1.3 | 1.7 | 4.8 |

* The relative fold change values of HpMpk1p were calculated by dividing induced phosphorylation levels with basal phosphorylation levels, which were measured in each strain background in Fig. 3A and B.
